# Exposure of *Bifidobacterium longum* subsp. *infantis* to Milk Oligosaccharides Increases Adhesion to Epithelial Cells and Induces a Substantial Transcriptional Response

**DOI:** 10.1371/journal.pone.0067224

**Published:** 2013-06-21

**Authors:** Devon W. Kavanaugh, John O’Callaghan, Ludovica F. Buttó, Helen Slattery, Jonathan Lane, Marguerite Clyne, Marian Kane, Lokesh Joshi, Rita M. Hickey

**Affiliations:** 1 Biosciences Department, Teagasc Food Research Centre, Moorepark, Fermoy, Cork, Ireland; 2 Glycoscience Group, National Centre for Biomedical Engineering Science, National University of Ireland, Galway, Ireland; 3 Department of Microbiology, University College Cork, Cork, Ireland; 4 Alimentary Pharmabiotic Centre, University College Cork, Cork, Ireland; 5 School of Medicine and Medical Science, University College Dublin, Belfield, Dublin, Ireland; University of Helsinki, Finland

## Abstract

In this study, we tested the hypothesis that milk oligosaccharides may contribute not only to selective growth of bifidobacteria, but also to their specific adhesive ability. Human milk oligosaccharides (3′sialyllactose and 6′sialyllactose) and a commercial prebiotic (Beneo Orafti P95; oligofructose) were assayed for their ability to promote adhesion of *Bifidobacterium longum* subsp. *infantis* ATCC 15697 to HT-29 and Caco-2 human intestinal cells. Treatment with the commercial prebiotic or 3′sialyllactose did not enhance adhesion. However, treatment with 6′sialyllactose resulted in increased adhesion (4.7 fold), while treatment with a mixture of 3′- and 6′-sialyllactose substantially increased adhesion (9.8 fold) to HT-29 intestinal cells. Microarray analyses were subsequently employed to investigate the transcriptional response of *B. longum* subsp. *infantis* to the different oligosaccharide treatments. This data correlated strongly with the observed changes in adhesion to HT-29 cells. The combination of 3′- and 6′-sialyllactose resulted in the greatest response at the genetic level (both in diversity and magnitude) followed by 6′sialyllactose, and 3′sialyllactose alone. The microarray data was further validated by means of real-time PCR. The current findings suggest that the increased adherence phenotype of *Bifidobacterium longum* subsp. *infantis* resulting from exposure to milk oligosaccharides is multi-faceted, involving transcription factors, chaperone proteins, adhesion-related proteins, and a glycoside hydrolase. This study gives additional insight into the role of milk oligosaccharides within the human intestine and the molecular mechanisms underpinning host-microbe interactions.

## Introduction

The microflora of the human gastrointestinal (GI) tract outnumber the cells of the human host by a factor of 10, with the bacterial population composed of up to 400 different species from 40–50 genera [1], [2]. Intestinal colonisation by commensal bacteria is associated with several beneficial outcomes to the host, including resistance to colonisation by pathogens, the provision of nutrients through degradation of non-digestible food components, the production of metabolites and short-chain fatty acids, and modulation of mucosal immunity, among others [2]. Many benefits to the host are associated with colonisation by bifidobacteria in particular [3] and therefore methods to increase the numbers of intestinal bifidobacteria have been actively pursued [3]. The two approaches most frequently employed are the delivery of live bacteria (probiotics) within a food source, or the use of specific carbohydrates or prebiotics (inulin, fructo- and galacto-oligosaccharides) which are known to survive gastric transit and are fermented in the colon by beneficial bacteria, thus promoting their growth [4].

Following birth, the neonatal intestinal tract is colonised predominantly by *Staphyloccocus*, *Streptococcus*, and *Enterobacteriaceae*, rapidly followed by a transition to obligate anaerobes, including *Bacteroides*, *Clostridium*, and *Bifidobacterium* [5]. The transition of the intestinal microflora during early life can be influenced by the source of nutrition. Human milk has evolved to become the optimal source of infant nutrition, containing various components that benefit the neonate including oligosaccharides, protein, and immune factors [6]. Breastfeeding is associated with higher counts of bifidobacteria in neonatal feces at one week of age, as well as typically lower counts of bacteroides, eubacteria, peptococci, veillonella, clostridia, and enterobacteria than formula-fed neonates [7], [8]. Human milk oligosaccharides (HMO) are believed to serve as a natural source of prebiotics for infants which stimulate the growth of bifidobacteria [9], [10]. Moreover, HMOs have also been shown to possess anti-adhesive effects that reduce the binding of pathogenic bacteria to the host cells [11]. Interestingly, human milk contains between 5 and 23 g/L of these oligosaccharides, with over 200 different HMO structures, which differ in their size, charge, and sequence [12]. The genome of *Bifidobacterium longum* subsp. *infantis* ATCC 15697 has been fully sequenced, allowing investigations into the genetic basis and molecular mechanisms underlying the adaptation of this strain to the intestine, including metabolism of HMOs. Many of its genes encode enzymes that are active on HMOs and in particular, a novel 43-kbp region dedicated to oligosaccharide utilization is present in the genome [13]. Research to date suggests that one of the primary roles of milk oligosaccharides is to act as a source of prebiotics, however, the first step in establishment of bacterial populations in the intestine is the adherence of the organisms in the gut environment.

Previously, Gonzalez *et al.*, (2008), have demonstrated that growth of *Bifidobacterium longum* in de-fatted human milk leads to the genetic up-regulation of putative type II glycoprotein binding fimbriae, which are implicated in attachment and colonisation [14]. Recently, Chichlowski *et al*. (2012), demonstrated that growth of *Bifidobacterium longum* subsp. *infantis* ATCC 15697 to mid-exponential phase on human milk oligosaccharides as the sole carbon source increased adherence to the HT-29 cell line [15]. As the majority of oligosaccharides in breast milk are able to traverse the GI tract and reach the colon undigested [16], [17], perhaps HMOs may contribute not only to selective growth of commensal bacteria, but also to their specific adhesive ability. The aim of the current study is to investigate the adhesion- and metabolic-related transcriptomic changes of *B. longum* subsp. *infantis* following transient exposure to milk oligosaccharides and to correlate the observed phenotypic changes with transcriptional changes as determined by microarray analysis.

## Materials and Methods

### Bacterial Strains and Culture Conditions

*Bifidobacterium longum* subsp. *infantis* ATCC 15697 was purchased from DSMZ (Germany), *Bifidobacterium longum* subsp. *infantis* ATCC 15702 was obtained from ATCC (Middlesex, UK), *Bifidobacterium longum* NCIMB 8809 and *Bifidobacterium angulatum* NCIMB 2236 from NCIMB (Aberdeen, Scotland), and *Lactobacillus reuteri* DPC 6100 from the Teagasc Food Research Centre culture collection, (Fermoy, Ireland). Strains were stored in deMan Rogosa Sharpe (MRS) [Difco, Sparks, MD, USA] broth containing 50% glycerol at −80°C and propagated twice in MRS media supplemented with L-cysteine (0.05% w/v) [Sigma, Steinheim, Germany] prior to use. Bacteria were routinely grown overnight at 37°C under anaerobic conditions generated using the Anaerocult A system (Merck, Dannstadt, Germany). All cultures were grown to an optical density (OD_600 nm_) of 0.4–0.5 to ensure entry into mid-logarithmic growth prior to use. Following the initial carbohydrate screening and lactose inhibition assays using *Bifidobacterium longum* subsp. *infantis* ATCC 15697, subsequent adhesion studies were done using mid-exponential phase cultures. To prepare mid-exponential phase cells, bacterial growth time was measured to determine the time of entry into the mid-exponential growth phase at which time the optical density was adjusted to 0.3, and the cultures were allowed to grow for a further 2 hours and used at an optical density (OD_600 nm_) ∼0.45–0.5 (corresponding to ∼5×10^7^ CFU/mL, ascertained by plate counts). The procedure was identical for the other bacterial strains tested.

### Bacterial Oligosaccharide Exposure Conditions

The time taken for *B. longum* subsp. *infantis* ATCC 15697 to enter the mid-exponential growth phase was determined by constructing growth curves by measuring the optical density at 600 nm (OD_600_) at intervals during growth. Bacteria were harvested in mid-exponential growth phase (18 hours) and the optical density was adjusted to an OD_600_ of 0.3, incubated for a further 2 hours and used at an optical density (OD_600 nm_) ∼0.45–0.5 (corresponding to approximately 5×10^7^ CFU/mL, ascertained by plate counts on MRS agar). The *B. longum* subsp. *infantis* cells were washed twice by centrifugation and resuspension in PBS, before final resuspension in McCoy’s 5A tissue culture media supplemented with 2% Fetal Bovine Serum (FBS). 1 mg/mL of either 6′sialyllactose, 3′sialyllactose, or a combination of the two (1 mg/ml each) was added and incubated for 3 hours at 37°C under anaerobic conditions. A control sample using tissue culture medium without oligosaccharide supplementation was also prepared. Bacteria were harvested by centrifugation (10,000×g, 8 minutes), the supernatants removed and pellets resuspended in RNAprotect (Qiagen, Hilden, Germany) for 10 minutes at room temperature followed by storage at −80°C prior to RNA extraction. As a control, non-supplemented tissue culture media was used. Additionally, exposures were replicated in the presence of 1 mg/ml lactose.

### Epithelial Cell Line Conditions

The HT-29 (human colonic adenocarcinoma) and Caco-2 cell lines were used as a model of the human intestinal epithelial layer. HT-29 and Caco-2 cells were routinely cultured in McCoy’s 5A media (10% FBS; 1% penicillin/streptomycin) and DMEM (10% FBS; 1% penicillin/streptomycin; 1% non-essential amino acids) (Sigma, Steinheim, Germany), respectively, in 75 cm^2^ tissue culture flasks at 37°C in a humidified 5% CO_2_ atmosphere. The cultures were passaged by detaching with trypsin when the cell growth had reached approximately 80% confluency. Cultures between passage numbers 15 and 18 (HT-29) and 20 to 24 (Caco-2) were used for adhesion studies. For adhesion assays, cells were seeded at a density of 2×10^5^ cells/mL into 12-well plates (Cellbind; Corning, New York, USA) and grown to late post-confluence as described by Coconnier *et al.* [18]. HT-29 and Caco-2 cells were used 21 days and 14 days post-confluence, respectively. Twenty-four hours prior to assay, media was substituted for McCoy’s 5A or DMEM (2% FBS, no antibiotics) for the appropriate cell line. Both flask and plate cultures were fed by replacing the culture medium with fresh medium every other day.

### Adhesion Assays

Bacterial strains were grown overnight and harvested at an optical density (OD_600_) between 0.4–0.5 (corresponding to ∼5×10^7^ CFU/mL, determined by plate counts) by centrifugation (4500 g, 8 min), washed twice in phosphate buffered saline (PBS; Sigma, Steinheim, Germany) and resuspended in McCoy’s 5A or DMEM tissue culture media (2% FBS; further referred to as non-supplemented media) supplemented with 3′sialyllactose, 6′sialyllactose (Carbosynth, Berkshire, UK), or Beneo Orafti P95 (oligofructose) (Beneo Orafti, Dublin, Ireland) at the concentrations indicated in the results section. Non-supplemented media was used as a control. The bacteria were exposed to the oligosaccharides at 37°C for the times indicated in the results section, washed once in PBS to remove the supplemented oligosaccharides, and resuspended in non-supplemented McCoy’s 5A/DMEM media prior to use in the assays.

Eukaryotic cells were washed twice with PBS, and 500 µl of the bacteria:media suspensions were added to the appropriate wells, corresponding to approximately 10–20 bacterial cells per cell. The number of viable cells per well was determined by detaching the cells with trypsin and counting in a Neubauer hemocytometer, and was about 3×10^6^ cells per well. Bacterial exposure to eukaryotic cells was for 2 hours at 37°C under anaerobic conditions. The exposure conditions were optimized to ensure maximal viability of bacterial and mammalian cells, with the viability of the eukaryotic cells remaining at 96–98% throughout the assays. After two hours, the wells were washed three times with PBS to remove any non-adherent bacteria and lysed with 500 µl of PBS containing 0.1% Triton X100; (Sigma, Steinheim, Germany) for 30 min at 37°C on a shaking platform at 110 agitations per minute to ensure maximal recovery of viable bacterial cells. The lysates were serially diluted and enumerated by spread-plating on MRS plates. Aliquots of the experimental inocula were retained, diluted and plated to determine original CFU/mL. The results were expressed as adherent bacteria as a percentage of the original inoculum, thereby accounting for variations in the original inocula between groups. Percentage adherent = [CFU/mL of recovered adherent bacteria ÷ CFU/mL of inoculum]×100. Each adhesion assay was conducted independently in triplicate over 3 successive passages of intestinal cells.

During carbohydrate exposure, bacteria were harvested and plated for CFU/mL on MRS agar at the following timepoints: 0, 1.5, and 3 hrs exposure in order to determine if bacterial numbers increased over the duration of the assay. In addition, the effect of longer incubation times was examined by harvesting an overnight culture and adjusting the cell density to an OD_600_ = 1.0 (∼2×10^8^ CFU/mL). The cells were then washed in PBS and resuspended in McCoy’s 5A tissue culture media supplemented with 2% FBS. One millilitre of the bacterial suspension was added to 3 mL of either non-supplemented tissue culture media or media supplemented with 6′sialyllactose (final concentration of 1 mg/mL). Aliquots were removed at 3, 6, 9, and 24 hours to determine CFU/mL by decimal dilutions and plating on MRS agar.

To determine if the oligosaccharide concentration had decreased during exposure to the bacteria, aliquots of the original and spent media following exposure were analysed by High pH Anion Exchange Chromatography with Pulsed Amperometric Detection (HPAEC-PAD) using a Dionex ICS-3000 system equipped with an ED40 electrochemical detector with a gold cell and LC30 chromatography oven. A CarboPac PA100 (250×4 mm) column protected with a CarboPac PA100 (50×4 mm) guard column was used. The mobile phase was 100 mM NaOH, 25 mM NaAc (0–40 min), 100 mM NaOH, 250 mM NaAc (40–40.01 min), and 100 mM NaOH, 25 mM NaAc (40.01–50 min). The limit of detection was 10 parts per million for this assay.

### RNA Isolation and Microarray Hybridisation

Bacterial RNA was isolated using the RNA isolate mini kit (Bioline, London, UK) with modifications. Bacterial aliquots were thawed on ice and pelleted at 5,000×g for 10 minutes. The supernatant (RNAprotect) was removed and the bacterial pellets resuspended in 100 µL TE buffer (50 mg/mL lysozyme, 1000 units/mL mutanolysin; Sigma, Poole, Dorset, UK) at 37°C for 30 minutes. Bacteria were then added to 2 mL screw-cap vials containing glass beads (Sigma) (filled to 0.25 mL mark) and 800 µL lysis buffer (Bioline kit). The cells were disrupted by bead-beating (Mini-Beadbeater-16; BioSpec Products Ltd, Bartlesville, USA) for 3 intervals of 20 seconds and placed on ice between each interval to cool the samples. The tubes were centrifuged at 10,000×g for 10 minutes at 4°C to collect glass beads and cell debris. Supernatants were removed and processed according to the manufacturer’s instructions commencing at the post-lysis step. RNA quantity and quality was assessed on a NanoDrop 1000 (Wilmington, DE, USA). Only samples with 260/280 nm readings >1.9, and 230/260 nm readings ≥1.00 were used. Sample quality was further assessed by agarose gel electrophoresis (NorthernMax buffer, Bioline, London, UK). RNA samples were subsequently shipped to IMGM laboratories (Martinsried, Germany) for labelling and DNA microarray analysis. Prior to their use in the microarray experiments the quality of the RNA was assessed using an Agilent Bioanalyser (RNA600 Chip) to ensure that degradation of the samples had not occurred during transit. All samples used for microarray analysis had an RNA integrity number (RIN) of 10, indicating that the RNA was of excellent quality.

### Microarray Analysis

The RNA was labelled for analysis using an RT-IVT protocol. Prior to labelling, the RNA was spiked with synthetic polyadenylated transcripts (Agilent spike-in controls). For each RT-IVT reaction, 500 ng of RNA was used and labelled as follows. The spiked total RNA was reverse transcribed into cDNA using random priming (Full Spectrum™ MultiStart Primers for T7 IVT, System Biosciences (SBI)) and then converted into labelled cRNA by *in-vitro* transcription (Quick-Amp Labelling Kit One-Color, Agilent Technologies) incorporating Cyanine-3-CTP. The manufacturers protocols were followed for both the reverse transcription and labelling steps.

The efficiency of labelling was determined both by using a NanoDrop analyser and by analysis on an Agilent 2100 Bioanalyser with a 6000 Nano LabChip Kit (Agilent Technologies, Co. Cork, Ireland). Only samples with cRNA yields >825 ng and with a specific activity of 9.0 pmol cyanine3 per microgram of cRNA were used for array analysis.

Following cRNA clean-up and quantification (NanoDrop), 600 ng of each Cyanine-3-labeled cRNA sample was fragmented and prepared for One-Color based hybridization (Gene Expression Hybridization Kit, Agilent Technologies, Co. Cork, Ireland). cRNA samples were hybridized at 65°C for 17 hrs on separate custom *Bifidobacterium* GE Microarrays (8×15K format).

Afterwards, microarrays were washed with increasing stringency using Gene Expression Wash Buffers (Agilent Technologies) followed by drying with acetonitrile. Fluorescent signal intensities were detected with Scan Control A.8.4.1 Software (Agilent Technologies) on the Agilent DNA Microarray Scanner and extracted from the images using Feature Extraction 10.7.3.1 Software (Agilent Technologies) and the design file 033172_D_F_20110831.xml.

### Analysis of Microarray Data

The raw microarray results were analysed using the Limma package of the R statistics suite. Background correction was performed by the normexp method with offset = 12. The inter-array normalisation method chosen was the Cyclic Loess normalisation algorithm. The array data were clustered using the heatmap2 package in R to identify any experimental samples deemed to be outliers. The fold-changes in gene expression were calculated as actual fold-change relative to the control samples. The cut-off for identifying genes of interest was a fold change of >2 and an uncorrected p-value of <0.05. As the majority of the differentially expressed genes identified from the microarray data were confirmed by real-time quantitative RT-PCR, adjusted P-values to correct for multiple sample errors were not taken into consideration as false detections would be identified by qPCR.

### qPCR Analysis

Complementary DNA (cDNA) was synthesized from 1 µg of RNA incubated with 3.2 µg of random hexamers, 0.5 µl of Transcript Reverse Transcriptase (Roche), 0.5 µl of Protector RNAse inhibitor, 1 mM dNTPs mix and 4 µl of Transcriptor RT Reaction Buffer (Roche), in a final volume of 20 µl. Template and random primers were incubated at 65°C for 10 min, followed by addition of the remaining components. The mix was incubated at 55°C for 30 min. Finally, Transcript Reverse Transcriptase was inactivated by heating to 85°C for 5 min.

PCR primers and probes were designed using the Universal Probe Library Assay Design Centre (Roche, West Sussex, UK). Primer sequences and probe combinations are listed in Supplementary Table S1.

16S rRNA and cysteinyl-tRNA synthetase were used as endogenous controls to correct for variability in the starting total RNA and provide a stable expression marker, against which relative levels of expression could be determined. Amplification reactions contained 2.5 µl of cDNA, 5 µl of 2X SensiMix II Probe Buffer (Bioline), 5 pmol/µl of each primer and probe mix and were brought up to a total of 10 µl by the addition of RNase-free water.

All reactions were performed in duplicate in 384-well plates on the LightCycler®480 System (Roche). Positive and negative controls were included in each run. Thermal cycling conditions applied were 95°C for 10 mins, [95°C for 10 seconds, 60°C for 45 seconds, and 72°C for 1 second to allow for fluorescence acquisition] (55 cycles), 40°C for 10 seconds as recommended by the manufacturer (Roche, West Sussex, UK). The 2^−ΔΔCt^ method [19] was employed to calculate relative changes in gene expression.

### Statistical Analysis

All adhesion studies were carried out on three separate occasions in triplicate. Results are presented as mean ± standard deviation of replicate experiments. Graphs were drawn using Microsoft Excel and the Student *t*-test and one-way ANOVA were used for pairwise or multiple comparisons, respectively, to determine statistically significant results, where p<0.05 was considered significant.

## Results

### Promotion of Bacterial Adhesion by Selected Carbohydrates

**Figure 1:**
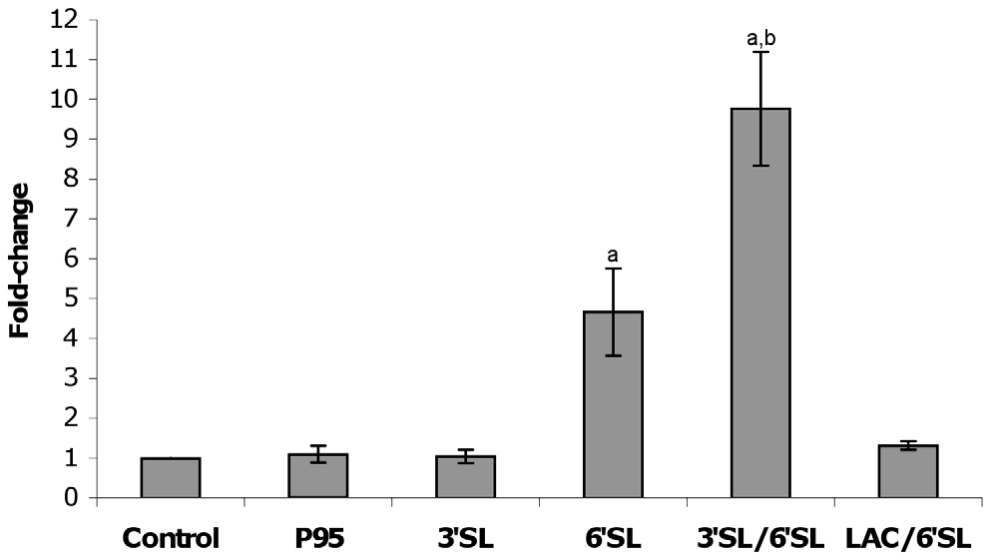
Screening oligosaccharides for their ability to influence adhesion of *B. longum* subsp. *infantis* ATCC 15697 to HT-29 cells. Abbreviations: P95 - Beneo Orafti P95; 3′SL - 3′sialyllactose; 6′SL –6′sialyllactose; Lac - lactose. Non-supplemented tissue culture media was used as control. Results are expressed as fold-change relative to control percent adhesion with error bars representing standard deviation. ^a^denotes significant difference in relation to control; ^b^denotes significance in relation to 6′SL group; p<0.0001.

*B. longum* subsp. *infantis* ATCC 15697 grown to mid-logarithmic phase were incubated separately with the two predominant milk oligosaccharides found in human and bovine milk (3′- and 6′-sialyllactose) or a commercial prebiotic (Beneo Orafti P95) for three hours at a concentration of 1 mg/ml, after which oligosaccharides were removed and their ability to adhere to HT-29 and Caco-2 cells was determined. Addition of 6′sialyllactose to mid-exponential cells resulted in a marked increase in adhesive ability, as represented by a 4.7-fold increase in adhesion of *B. longum* subsp. *infantis* ATCC 15697 to the HT-29 cells versus the control (p<0.0001). No change in adhesion of *B. longum* subsp. *infantis* ATCC 15697 to HT-29 cells was observed following incubation alone with 3′sialyllactose, Beneo Orafti P95 (Fig. 1) or lactose (data not shown).

When combinations of the structurally similar milk sugars were tested, simultaneous exposure of *B. longum* subsp. *infantis* ATCC 15697 to 3′sialyllactose and 6′sialyllactose (1 mg/mL each) produced an enhanced effect with an increase in percent adhesion of 9.8-fold over control (p<0.0001; Fig. 1). In contrast, pairing lactose with 6′sialyllactose completely abolished the ability of 6′sialyllactose to enhance bacterial adhesion.

Adhesion of *B. longum* subsp. *infantis* ATCC 15697 to Caco-2 cells following pre-treatment with oligosaccharides displayed a different response than that of the HT-29 cell line. 3-Sialyllactose pre-treatment resulted in the greatest effect upon bacterial adhesion (1.85-fold increase), while pre-treatment with 6′sialyllactose or the combination of 3′- and 6′-sialyllactose increased adhesion by 1.53-fold and 1.58-fold, respectively (p<0.01) (Fig. S1). Pre-treatment with lactose or oligofructose (P95) did not significantly affect bacterial adhesion to the Caco-2 cell line.

### Effect of 3′sialyllactose, 6′sialyllactose, Lactose and P95 on the Growth Characteristics of *B. longum* subsp. *infantis* ATCC 15697

Growth studies were carried out to determine if the screened carbohydrates were capable of promoting the growth of *B. longum* subsp. *infantis* ATCC 15697 under the experimental conditions used in the adhesion study. Lactose alone promoted the growth of the bacteria (data not shown) within the three hour incubation period. No change in growth rate was observed with 3′sialyllactose, 6′sialyllactose, oligofructose (P95), or the non-supplemented media. It was further confirmed that 6′sialyllactose did not affect growth of *B. longum* subsp. *infantis* following 3, 6, 9, and 24 hours of incubation in tissue culture media under identical conditions used in the adhesion assays (data not shown).

### The Effect of Bacterial Exposure on Oligosaccharide Concentrations

**Figure 2:**
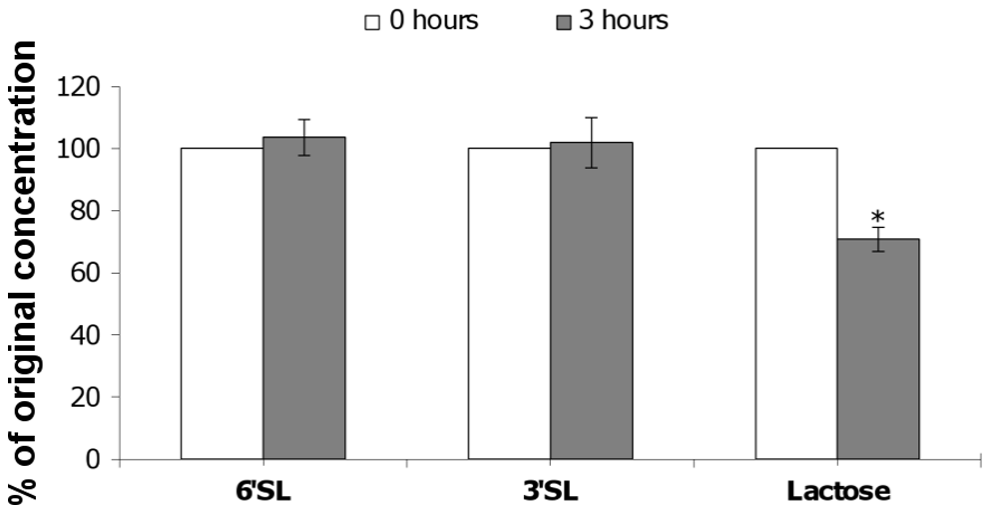
Bacterial influence on oligosaccharide concentrations during exposure. Aliquots of media prior to and following exposure with *B. longum* subsp. *infantis* ATCC 15697 for 3 hours were assessed by HPLC. Abbreviations: 6′SL - 6′Sialyllactose; 3′SL - 3′sialyllactose. Results are expressed as percentage of initial oligosaccharide concentration in non-exposed media for each treatment group with error bars representing standard deviation. *denotes p<0.005.

To assess whether the increased adhesion was associated with metabolism of the added oligosaccharides by the bacteria during the 3-hour exposure period, the oligosaccharide content of media before and after incubation with bacteria was determined by HPAEC-PAD (Fig. 2). No significant change was noted in the concentration of 6′sialyllactose following exposure at 1 mg/mL to the bacteria. The concentration of 6′sialyllactose increased by 3.7%, which was non-significant. When lactose and 6′sialyllactose were incubated with bacteria together (1 mg/mL each), a significant decrease of 29% in lactose concentration was detected (p = 0.0035), while 6′sialyllactose showed an increase of 5.9% (non-significant). Interestingly, the media transitioned from red to yellow during the 3-hour bacterial incubation in the presence of lactose (alone or in combination with 6′sialyllactose; data not shown) indicating acidification. Bacterial exposure to the combination of 3′sialyllactose and 6′sialyllactose (1 mg/mL each) resulted in non-significant increases in both 3′sialyllactose (1.9%) and 6′sialyllactose (2.6%). These results confirm that the two oligosaccharides, 3′sialyllactose and 6′sialyllactose, were not significantly catabolised during bacterial exposure, while lactose was preferentially consumed.

### Influence of Oligosaccharide Concentration and Exposure Time on Bacterial Adhesion to HT-29 cells

**Figure 3:**
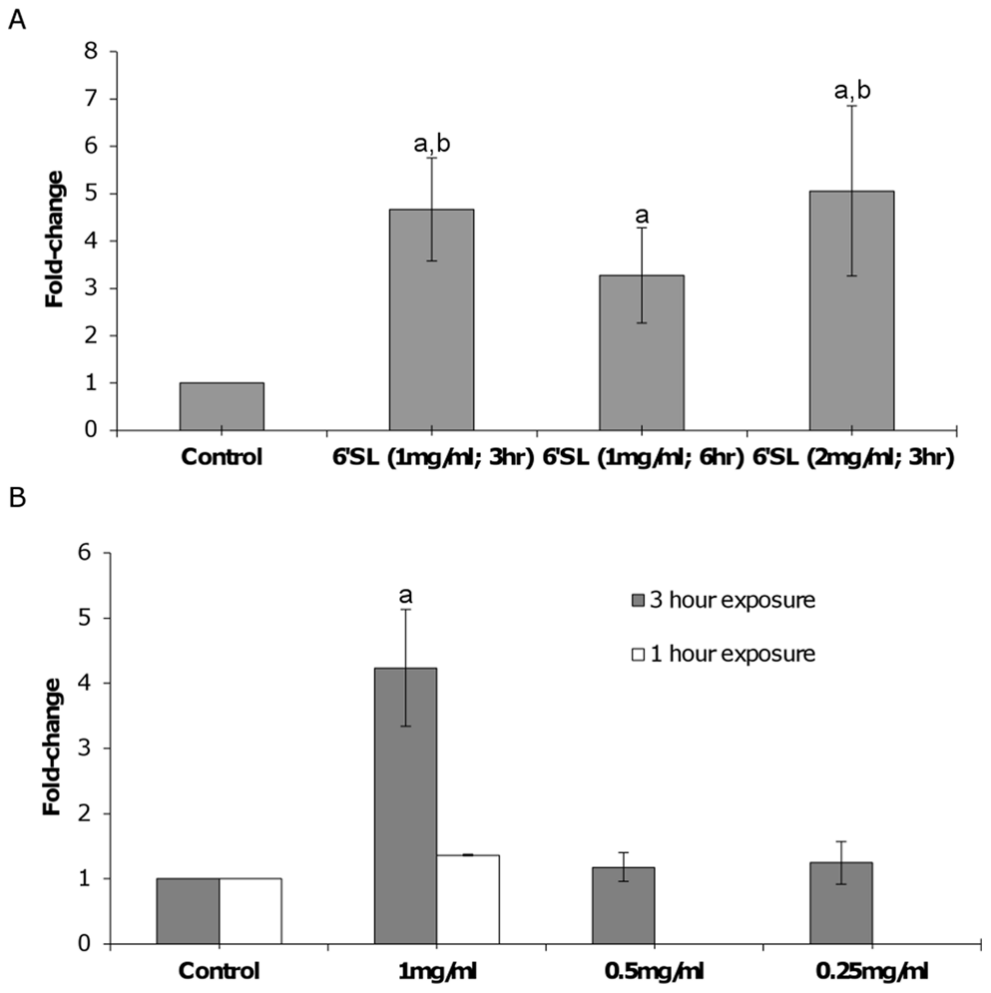
Dose-response of *B. longum* subsp. *infantis* ATCC 15697 to 6′sialyllactose and subsequent adherence to HT-29 cells. (A) Bacterial exposure to original conditions (1 mg/ml; 3 hours), increased duration (1 mg/ml; 6 hours) or increased concentration (2 mg/ml; 1 hour) in comparison to non-treated control bacteria. (B) Exposure to a gradient of 6′sialyllactose was assayed over 3 hours (gray shading) or control and 1 mg/ml for 1 hour exposure (white shading). Results are expressed as fold-change in percent adhesion in relation to the respective control ± standard deviation. ^a^denotes significance in relation to the control; ^b^denotes significance in relation to 6′SL (1 mg/ml; 6 hr) treatment; p<0.0001.

To determine if the effect of 6′sialyllactose on the adhesion of *B. longum* subsp. *infantis* ATCC 15697 could be enhanced, the effects of increased exposure duration (1 mg/ml, 6 hours) or exposure concentration (2 mg/ml, 3 hours) on adhesion of bacterial cells were examined. Each treatment group resulted in a significant increase in percent adhesion in relation to the control (4.7-fold, p<0.0001 [1 mg/ml; 3 hours]; 3.3-fold, p<0.0001 [1 mg/ml; 6 hours]; 5.1-fold, p<0.0001 [2 mg/ml; 3 hours]). Neither increasing the concentration of 6′sialyllactose nor the length of exposure significantly increased adhesion to HT-29 cells in comparison to the original exposure conditions (1 mg/mL; 3 hour incubation; Fig. 3A). Given the current findings, an incubation time of 3 hours appears to produce the maximal increase in adhesion of the bacteria to the HT-29 cells.

As increasing the concentration of 6′sialyllactose did not significantly increase bacterial adhesion beyond that previously achieved, lower concentrations were investigated. Subsequent assays were performed using lower concentrations of 6′sialyllactose (0.25 and 0.50 mg/mL) with an exposure time of 3 hours and these concentrations did not significantly alter the adhesion of the bacteria in relation to the control, while exposure to 1 mg/ml continued to demonstrate a significant increase in adhesion (4.2 fold; p<0.0001) (Fig. 3B). Additionally, no increase in adhesion was noted after 1-hour exposure of bacteria to 6′sialyllactose (1 mg/mL) (Fig. 3B).

### Effect of Trypsin-treatment on Adhesion of *B. longum* subsp. *infantis* ATCC 15697 to HT-29 Cells Following Oligosaccharide Exposure

**Figure 4:**
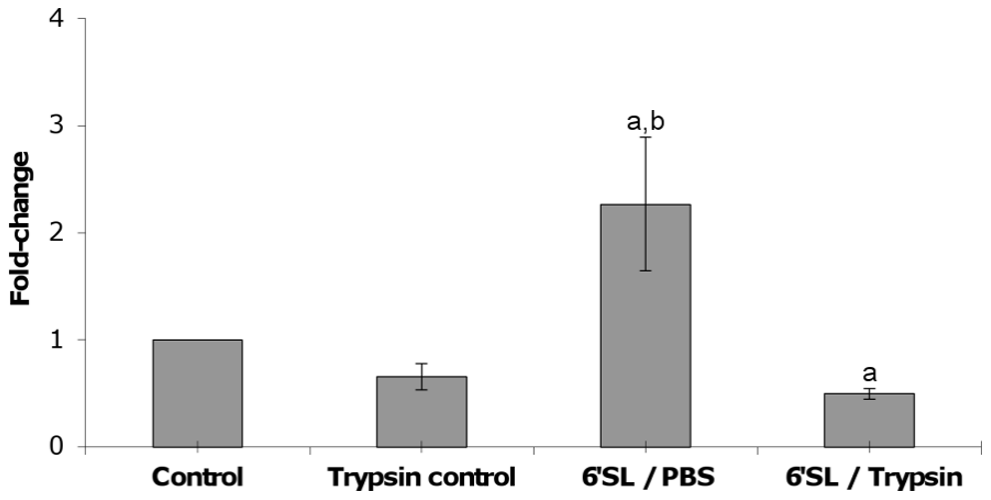
Effects of trypsin treatment on adhesion of *B. longum* subsp. *infantis* ATCC 15697 to HT-29 cells following exposure to 6′sialyllactose (1 mg/ml, 3 hours). Results are expressed as fold-change in percent adhesion in relation to the control with error bars representing standard deviation. ^a^denotes significance in relation to the control, p<0.05; ^b^denotes significance in relation to Trypsin control and 6′SL/Trypsin groups; p = 0.0001.

To explore whether the increased adhesion of the bacteria to HT-29 cells was mediated by a surface protein, *B. longum* subsp. *infantis* cells were treated with trypsin in PBS or PBS alone (1 hour) following 6′sialyllactose exposure (1 mg/ml, 3 hours), and used subsequently in adhesion assays. The results indicate that trypsin-treatment following 6′sialyllactose exposure significantly reduces the adhesive percentage of the bacteria to HT-29 cells compared to the control (p<0.05), while 6′sialyllactose exposure followed by incubation in PBS for 1 hour still resulted in increased adhesion (p<0.05) (Fig. 4). Trypsin treatment was carried out on a control group following exposure to non-supplemented media for 3 hours, resulting in a non-significant reduction in the adhesive percentage versus the untreated control (p = 0.0561). Furthermore, comparison among the 6′sialyllactose-treated groups indicates that enzymatic treatment resulted in a significant reduction in adhesion to the HT-29 cells (p<0.0001). The results provide support for the involvement of a surface protein or proteins in the normal adhesion of *B. longum* subsp. *infantis* ATCC 15697 to HT-29 cells and the increased adhesion following exposure to 6′sialyllactose.

### Increased Adhesion Response is Unique to *B. longum* subsp. *infantis* ATCC 15697

To assess whether 6′sialyllactose could promote the adhesion of other commensal strains, adhesion studies were performed under identical conditions with three different strains of bifidobacteria and a *Lactobacillus* strain. No significant increases in adhesion were observed for any of the other strains tested (Table 1), suggesting that the observed effect was species/strain-specific. The percentage adhesion of the bifidobacteria strains was much lower than the *Lactobacillus reuteri* (DPC 6100) (0.09–2.29% of the inoculum versus 12.0%, respectively).

**Table 1:** Screen of commensal strains for increased adherence to HT-29 cells following exposure to 6′sialyllactose.

	*B. longum* subsp.*infantis* ATCC 15697	*B. longum* subsp. *infantis*ATCC 15702	*B. longum*NCIMB 8809	*B. angulatum*NCIMB 2236	*L.reuteri*DPC 6100
Control (%Adherent)	0.40%±0.18	0.094%±0.019	2.29%±0.38	1.14%±0.066	12.00%±2.30
6′SL-exposed (%Adherent)	1.81%±0.71	0.14%±0.050	1.61%±0.30	0.90%±0.53	11.57%±1.20
Fold-change	4.53	1.47	0.70	0.79	0.96

### Gene Expression Analysis by DNA Microarray

**Figure 5:**
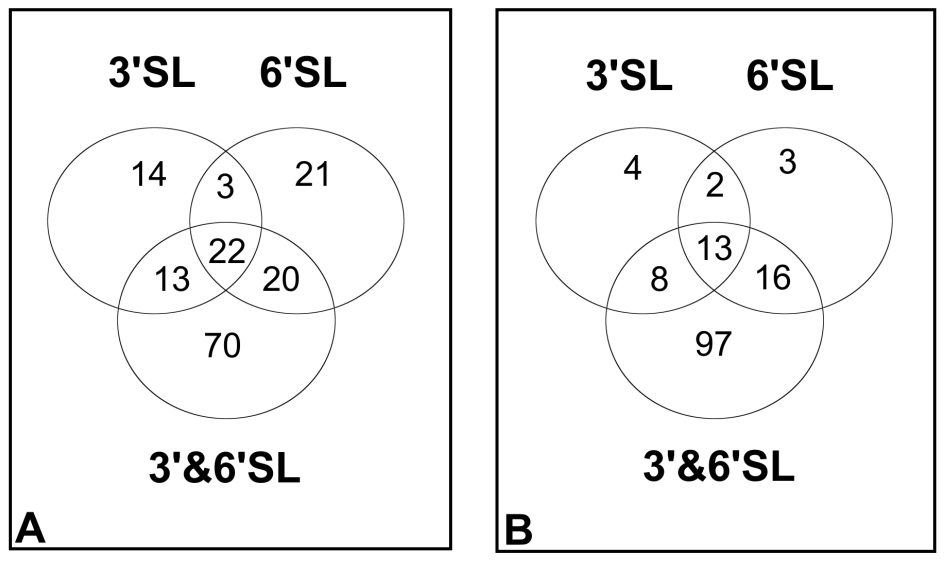
Differentially expressed transcripts following exposure to the three oligosaccharide treatments. (A) up-regulated transcripts (B) down-regulated transcripts. The cut-off point for inclusion was a p-value <0.05.

The overall numbers of genes differentially expressed following the experimental treatments are shown by Venn diagram (Fig. 5). It can be seen from this analysis that while 3′- and 6′-sialyllactose alone induced numerous differentially expressed transcripts (DETs) in comparison to control media, that there was a very substantial synergistic effect observed when the *B. longum* subsp. *infantis* ATCC 15697 cells were treated with a mixture of 3′- and 6′-sialyllactose. Following exposure to the mixed oligosaccharides, a total of 135 DETs were up-regulated in comparison to 66 following treatment with 6′sialyllactose and 52 following treatment with 3′sialyllactose (Fig. 5). A relatively low number of genes (22) were differentially regulated by all three treatments (Table 2). As well as inducing the highest number of DETs following upon oligosaccharide treatment, the oligosaccharide mixture induced the highest number of treatment specific genes (70) compared to 21 for 6′sialyllactose and 14 for 3′sialyllactose (Fig. 5). The genes with altered levels of transcription are described in Tables S2, S3, and S4.

**Table 2:** Up-regulated genes common to all three oligosaccharide treatments.

		3′SL		6′SL		3′+6′SL	
Locus_tag	Gene Description	FC	pval	FC	pval	FC	pval
Blon_0029	Ferritin, Dps family protein	1.67	0.0002	1.72	0.0002	2.79	0.0002
Blon_0036	FAD-dependent pyridine nucleotide-disulphide oxidoreductase	1.31	0.017	1.26	0.036	1.78	0.0001
Blon_0392	Cation efflux protein	1.66	0.006	1.84	0.0001	3.04	0.00001
Blon_0460	Binding-protein-dependent transport systems inner membrane component	1.32	0.037	1.38	0.02	1.81	0.001
Blon_0617	Glutamate-cysteine ligase, GCS2	1.32	0.022	1.63	0.0009	1.37	0.014
Blon_0758	Glutaredoxin-like protein	1.45	0.004	1.45	0.004	1.99	0.0007
Blon_0759	ABC transporter related	1.42	0.029	1.37	0.05	1.93	0.0007
Blon_0902	Initiation factor 3	1.65	0.005	1.59	0.008	1.43	0.012
Blon_0947	helix-turn-helix domain protein	1.32	0.01	1.35	0.007	1.54	0.0002
Blon_0948	hypothetical protein	1.3	0.02	1.3	0.02	1.5	0.003
Blon_0992	hypothetical protein	1.43	0.021	1.98	0.004	2.47	0.002
Blon_1687	TfoX, C-terminal domain protein	1.56	0.009	1.93	0.0006	2.26	0.001
Blon_1688	transcription activator, effector binding	1.78	0.0005	2.35	0.00002	2.88	0.00006
Blon_1950	hypothetical protein	1.26	0.01	1.2	0.03	1.35	0.001
Blon_1951	UMUC domain protein DNA-repair protein	1.28	0.012	1.22	0.03	1.41	0.001
Blon_2191	ribose 5-phosphate isomerase	1.23	0.025	1.25	0.016	1.58	0.001
Blon_2370	glycerophosphoryl diester phosphodiesterase	1.71	0.0002	1.83	0.00008	2.09	0.00001
Blon_2371	Glutamate–tRNA ligase	1.45	0.002	1.75	0.0001	2.29	0.000005
Blon_2372	ATPase AAA-2 domain protein	1.7	0.0002	1.88	0.00004	2.45	0.000004
dnaK	chaperone protein DnaK	1.44	0.002	1.57	0.0005	1.97	0.00006
groEL	chaperonin GroEL	1.33	0.02	1.37	0.015	2.11	0.0022
recA	recA protein	1.19	0.044	1.26	0.014	1.21	0.027

3′SL –3′sialyllactose; 6′SL –6′sialyllactose; 3′+6′SL – combined treatment of 3′- and 6′-sialyllactose; FC – fold change; pval – p-value.

Given the effects of oligosaccharide treatment on adhesion, it is probable that only those genes that are up-regulated by more than one oligosaccharide treatment are central to the adhesive phenotype and may be considered the core component of the oligosaccharide response. The total number of core genes is 55, and of those, the 42 up-regulated genes shared between 6′sialyllactose and the 3′- and 6′-sialyllactose mixture (Fig. 5) are of particular interest as these treatments directly led to an increase in adhesion to HT-29 cells (Fig. 1). This group of 42 genes is listed in Table S5, where the function assigned to each (if known) is also stated.

**Figure 6:**
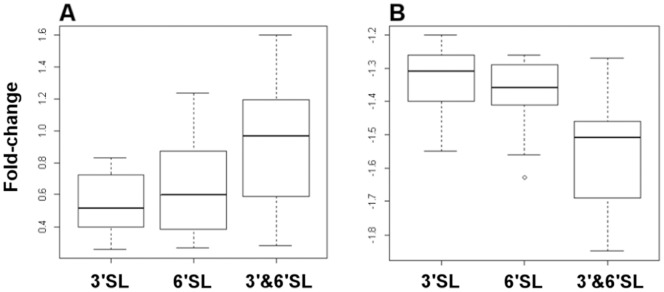
Boxplot representation of the fold-change in expression for the genes up-regulated (A) and down-regulated (B) by all three treatments.

Aside from the qualitative changes observed for gene expression, the quantitative aspect of the transcriptional response is also important. The synergistic effect of the oligosaccharide mixture on adhesion correlated strongly with a higher level of gene transcription induced by the mixed oligosaccharides. We have used boxplot type plots to represent the overall transcript levels for the 22 commonly up-regulated genes (Fig. 6a) and from this plot it may be seen that the median value for gene transcription following each treatment correlated extremely well with the observed effects on adhesion.

The final set of genes of interest includes those genes whose up-regulation was unique to either 6′sialyllactose or 3′ and 6′sialyllactose treatment. This group contains 21 genes specific to 6′sialyllactose treatment and 70 genes specific to the mixed 3′- and 6′-sialyllactose treatment (Fig. 5). Finally, we examined the expression profile of all of the genes up-regulated by the 3′- and 6′-sialyllactose mixture based on their position in the Venn diagram. Of the 4 treatment groups, the genes common to all three treatment groups (22) had the highest transcript level followed by those genes commonly up-regulated by 6′sialyllactose and the mixture group ([6′SL]+[3′&6′SL]). The genes common to the 3′sialyllactose treatment and the 3′- and 6′-sialyllactose mixture ([3′SL]+[3′&6′SL]) and, lastly, those unique to the combination of 3′- and 6′-sialyllactose, had the lowest levels of transcription (Fig. S2). From this analysis it is clear that the genes most significant are those that are up-regulated by all treatments.

Our detailed analysis of the transcriptional response has identified two significant groups of genes that are likely responsible for the enhanced adhesion response which are those common to all 3 treatments (Table 2) and those common to the 6′sialyllactose and the 3′- and 6′-sialyllactose mixtures (Table S5).

The number of genes for which expression was reduced in response to oligosaccharide treatment was roughly similar to the number of up-regulated genes. However, in general, the magnitude of the changes observed was lower than that for the up-regulated genes. The 13 genes down-regulated in common to all three treatments (Table 3) were down-regulated in inverse proportion to the observed adhesion effects, with the extent of down-regulation being highest for the cells treated with the oligosaccharide mixture (Fig 6b). Unlike the up-regulated transcripts, the majority of those genes that were down-regulated were associated with sugar transport and metabolism. Among the down-regulated genes that would be central to sugar metabolism are two transcriptional regulators (blon_0573 and blon_0879) from the ROK family of proteins. Both of these genes appear to be transcriptional repressors and their down-regulation is likely to result in the up-regulation of the genes that they regulate. Interestingly, one of the genes down-regulated by both 6′sialyllactose and the 3′- and 6′-sialyllactose mixture was an exo-alpha sialidase that is located in a large cluster of genes (blon_2331-blon_2361) that are involved in sugar metabolism. Following a closer examination of the expression patterns of milk oligosaccharide metabolism genes, it is clear that the majority of them are down-regulated slightly following treatment with 6′-sialyllactose and down-regulated more so by exposure to the mixture of 3′- and 6′-sialyllactose (Figs. S3 and S4).

**Table 3:** Down-regulated genes common to all treatments.

		3′SL		6′SL		3′+6′SL	
Locus tag	Gene Description	FC	pval	FC	pval	FC	pval
Blon_0335	trc regulator merR	−1.20	0.0400	−1.28	0.0100	−1.29	0.0060
Blon_0505	Hypothetical protein	−1.26	0.0200	−1.26	0.0190	−1.27	0.0100
Blon_0644	ROK family protein	−1.29	0.0400	−1.36	0.0200	−1.65	0.0080
Blon_0645	N-acetylglucosamine-6-phosphate deacetylase	−1.38	0.0300	−1.36	0.0300	−1.69	0.0030
Blon_0790	Proteinase inhibitor	−1.25	0.0500	−1.28	0.0300	−1.46	0.0040
Blon_0884	Extracellular solute binding protein	−1.31	0.0200	−1.29	0.0300	−1.47	0.0500
Blon_0885	Binding protein dependent sugar transport inner membrane protein	−1.25	0.0200	−1.40	0.0020	−1.50	0.0000
Blon_2174	Hypothetical protein	−1.40	0.0200	−1.55	0.0060	−1.75	0.0020
Blon_2175	Solute transport	−1.35	0.0300	−1.56	0.0030	−1.43	0.0100
Blon_2176	Solute transport	−1.46	0.0060	−1.63	0.0010	−1.51	0.0020
Blon_2341	Hypothetical protein	−1.29	0.0190	−1.33	0.0100	−1.73	0.0020
Blon_2379	Transport system inner membrane protein	−1.55	0.0070	−1.41	0.0200	−1.85	0.0020
Blon_2380	Sugar transport system solute binding protein	−1.51	0.0030	−1.38	0.0100	−1.53	0.0007

3′SL –3′sialyllactose; 6′SL –6′sialyllactose; 3′+6′SL – combined treatment of 3′- and 6′-sialyllactose; FC – fold change; pval – p-value.

### Gene Expression Validation by qPCR

**Figure 7:**
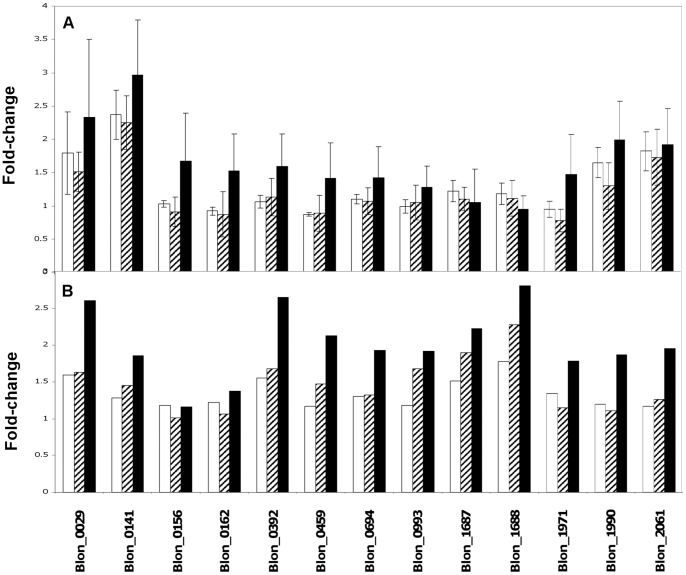
Transcript levels for thirteen (13) selected genes as determined by qPCR (A) and microarray analysis (B). 3′sialyllactose (white shading); 6′sialyllactose (black and white diagonal shading); 3′- and 6′-sialyllactose mixture (black shading). Blon_0029– Ferritin; Blon_0141 - Chaperonin protein DnaK; Blon_0156 - TadE family protein; Blon_0162– Sortase; Blon_0392 - Cation efflux protein;Blon_0459 - Glycoside hydrolase, family 20; Blon_0694– GroEL; Blon_0993 - Magnesium-translocating P-type ATPase; Blon_1687 - TfoX, C-terminal domain protein; Blon_1688 - Transcription activator, effector binding; Blon_1971 - High-affinity zinc ABC transporter; Blon_1990 - Glycine dehydrogenase; Blon_2061– Extracellular solute binding protein.

The genes of interest identified by DNA microarray were further validated through the use of real-time PCR. In addition to those selected from the array analysis, we included the sortase and tadE pilus in the qPCR screening, as these genes were previously reported to be significant contributors to the adhesive potential of different strains of bifidobacteria [20], [21] (Fig. 7). The 16S rRNA gene target produced a stable level of expression against which the relative expression of the selected gene targets could be assessed.

Two genes, Blon_1687 and Blon_1688, were found to be significantly up-regulated in the DNA microarray analysis, but were not validated through qPCR, as their levels of expression were nearly identical to those of the 16S rRNA control. Interestingly, four genes associated with bacterial adhesion were up-regulated following bacterial exposure to the combination of 3′- and 6′-sialyllactose; i) DPS-ferritin (Blon_0029); ii) GroEL (Blon_0694); iii) DnaK (Blon_0141); and iv) Sortase (Blon_0162) (Fig. 7). When bacterial oligosaccharide exposures were performed in the presence of lactose, expression of each of the selected genes of interest was similar to that of the control (data not shown).

## Discussion

By one month of age, bifidobacteria are the predominant fecal bacteria in neonates [7], with several strains demonstrating the ability to utilize the abundant supply of breast milk oligosaccharides as a carbon source [22], [23]. *Bifidobacterium longum* subsp. *infantis* ATCC 15697 is the archetypical human milk oligosaccharide consumer in the infant GI tract [24] and has previously been demonstrated to display an adherent phenotype following growth on human milk oligosaccharides in *in vitro* models [15]. However, the current study is the first to examine the effect of milk oligosaccharide treatment on the adhesive capacity of *B. longum* subsp. *infantis* ATCC 15697 and to identify specific transcriptional responses associated with an oligosaccharide-induced adherent phenotype.

While our findings were similar to that of Chichlowski *et al.*, the focus of our study was upon brief exposure (3 hours) to specific HMOs, as opposed to mid-exponential growth (48 hours) on a pool of HMOs [15]. Additionally, the current results were obtained through enumeration of live colonies by plating, compared to the methodology of qPCR. Bacterial adhesion to intestinal epithelial cells is strain- and species-specific [25]–[27]. Typically, the adhesive proportion of bifidobacteria, particularly *B. infantis* and *B. longum*, to Caco-2 and HT-29 cell lines is low (0–5%), with the exceptions of *B. bifidum*, *B. adolescentis* and *B. animalis* subsp. *lactis* Bb12 which display a greater degree of adhesion (10–30%) [25], [26], [28]–[33]. The current findings are in agreement with the previous studies in terms of percentage adherence and adherent bacteria/100 mammalian cells.

In agreement with the results of Chichlowski *et al.*, our results demonstrate that exposure of *B. longum* subsp. *infantis* ATCC 15697 to 3′SL, 6′SL, or a combination of the two, resulted in approximately 1.5–1.8-fold increased adhesion to Caco-2 cells and an increase in adhesion to HT-29 cells (3-4-fold increase following growth on a pool of HMOs versus 4-10-fold increase observed in the current study), though independent of growth upon the supplemented oligosaccharides. Confluent and fully-differentiated Caco-2 cells are similar to small intestine-like enterocytes [34], while HT-29 cells are colonic adenocarcinoma epithelial cells remaining undifferentiated (95%) in the post-confluent state [35]. Accordingly, the HT-29 model may better represent the *in vivo* immature intestinal environment of a neonate, as infant-type bifidobacteria encounter milk oligosaccharides in the colon [36], a destination in which sialylated milk oligosaccharides may be enriched [37]. Further *in vivo* colonisation studies are necessary to validate the model-specific effects observed.

In the current study, increased adhesion conferred by exposure to HMOs was not accompanied by a change in oligosaccharide content in the spent culture supernatant, indicating that under the given conditions, 3′- and 6′-sialyllactose were not catabolised. Additionally, it has previously been reported that semi-synthetic media supplemented with human milk oligosaccharides at less than 0.8% (8 mg/mL) resulted in only minimal growth of *B. longum* subsp. *infantis* ATCC 15697 [38], indicating that the concentrations used in the current study were not sufficient to stimulate growth. It is clear from analysis of both bacterial growth and the oligosaccharide content of the assay medium that the oligosaccharides did not confer a growth advantage to the bacteria under the conditions used in this study, thereby eliminating the possibility that the increased adhesion was due to an increase in the bacterial numbers during the assay.

While *in vivo* experiments are ideal, the implementation of *in vitro* cell lines, such as HT-29 and Caco-2, prove valuable. Though the two cell lines are not completely representative of *in vivo* conditions, they provide a tightly-controlled environment in which to assay the ability of bacterial strains to adhere to differentiated and non-differentiated enterocytes. Though the intestinal cell lines used do not produce the mucus layer that is typically present in *in vivo* conditions, the mucin producing cell lines currently available do not produce a mucus layer specifically representative of the large intestine or colon, while primary cell cultures present difficulties in reproducibility [39]. Furthermore, there exist situations wherein the mucus layer has not yet fully developed or becomes disrupted. Under these circumstances, adhesion of intestinal bacteria to colonocytes becomes an issue. In the given scenario, it is of advantage to the host to promote or accommodate colonisation by beneficial bacteria, as their colonisation will negate the effects of invasion by other potentially pro-inflammatory species.

It is important to remember that the *in vivo* exposure of intestinal bacteria to oligosaccharides present in the digesta will always take the form of exposure to a mixture of different oligosaccharides. The synergistic effect between the oligosaccharides reported here is evidence that *B. longum* subsp. *infantis* produces a more effective adhesion phenotype under conditions that better represent the intestinal milieu. While 3′sialyllactose and 6′sialyllactose are identical in molecular weight and contain the same functional groups, a marked difference in adhesion-promoting ability exists between the two, which infers a specific response to α-2,6-linked sialic acid. Furthermore, the presence of lactose completely abolished the increased adhesion phenotype of *B. longum* subsp. *infantis* ATCC 15697 to the HT-29 cell line. As reported by Garrido *et al.*, [40], growth of *Bifidobacterium longum* subsp. *infantis* ATCC 15697 on HMO as a sole carbon source, resulted in the expression of specific proteins that bind and import HMO isomers, but were also able to bind mucin and blood group glycans. If this finding is considered along with the fact that bacterial treatment with trypsin is accompanied by a significant reduction in adhesion, bacterial exposure to the milk oligosaccharide, 6′sialyllactose, may contribute to increased bacterial adhesion to the HT-29 intestinal model through expression of a proteinaceous surface receptor [41].

We observed a strong correlation between the number of differentially expressed transcripts following each oligosaccharide treatment and the observed increase in the adhesive potential of *B. longum* subsp. *infantis*. The use of Venn diagrams allowed us to identify genes that were induced by all of the treatments along with those genes induced specifically by certain treatments, thereby allowing for the separation of the transcriptional responses to oligosaccharide treatments into two categories. *B. longum* subsp. *infantis* mounts what appears to be a general response to oligosaccharide exposure, wherein, 22 genes are induced in common to all three oligosaccharide treatments. It is clear that the magnitude of change in the transcript level for these genes is important in developing the adhesive response, as there was a strong correlation between transcript expression level and adhesion to the HT-29 cell line. A similar trend was observed for the 20 genes co-induced by the 6′sialyllactose treatment and the oligosaccharide mixture and these genes may be considered as being more specific to the adhesion process as they were only up-regulated by the treatments that enhanced adhesion, with the extent of the increase in transcription correlating well with the changes in adherence.

It appears that the contribution of the individual genes to epithelial adherence is greater if they are differentially regulated by more than one treatment and these may be designated the core genes. The increased transcription of those genes that were unique to each treatment may reflect changes in cell processes occurring as a consequence of the up-regulation of the core genes rather than contributing to the adhesive process.

In terms of the enhanced HT-29 adhesion observed, the most significant gene identified was the DNA-binding protein-ferritin (dps-ferritin) locus. The dps-ferritin type family is a widely diverged family of proteins that has evolved to perform a range of functions other than DNA protection, including the formation of fine tangle pili in *Haemophilus ducreyii* [42] and possible roles in adhesion and protection against oxidative stress in *Helicobacter pylori* [43]. The function of this protein homolog (NapA) in *H. pylori* is of particular interest because *H. pylori* is effectively a commensal organism in the human gut and it is therefore quite probable that the dps-ferritin protein plays a similar role in *Bifidobacterium.* The close correlation observed between transcription of this dps-ferritin gene and the increased adhesion support the assumption that it is involved in virulence or adhesion. Members of the dps-ferritin family also have a role in stress response, particularly towards oxidative stress, and it has been demonstrated for *Salmonella enterocolitica* and *Listeria monocytogenes* that the dps-ferritin stress resistance is associated with increased virulence [44], [45].

GroEL is a well characterised heat shock protein similar both in structure and function to the eukaryotic Hsp60. GroEL is virtually ubiquitous in prokaryotes, being present in >90% of genomes examined [46] and is also present in bifidobacteria [47]. The most important function of groEL is in its role as a molecular chaperone wherein it acts to ensure that certain proteins are folded to the correct tertiary structure. In its protein-folding role, groEL requires the co-chaperonin groES in equimolar quantities and in *Bifidobacterium breve* UCC2003 groEL and groES are transcribed at similar levels in response to heat shock even though they are located in different regions of the genome. It is noticeable, however, that the levels of groES transcript induced in our studies did not correlate with those of groEL and this may indicate that the excess groEL may fulfil a role that is independent of groES. Furthermore, there was no evidence that groEL was co-transcribed with the contiguous cold-shock gene cspA (blon_0693) in this study unlike the case for other bifidobacteria [47], as transcript levels for cspA were lower following all treatments. These observations may indicate that regulation of groEL mRNA levels occurs by means of decreased RNA turnover rather than increased transcription levels and may suggest a role for groEL in adhesion that is independent of its chaperone function. Surface expression of the groEL protein has been previously shown in several pathogens and has been implicated in attachment and/or immunomodulatory activities [48]–[52]. Surface expression of groEL has also been reported for the probiotic *Lactobacillus johnsonii* La1 (NCC 533), and was demonstrated to bind to both mucins and epithelial cells, as well as possessing the ability to aggregate *Helicobacter pylori* [52]. In this study, increased levels of groEL transcript followed all three treatments, with the greatest response following exposure to the mixture of 3′- and 6′-sialyllactose which correlated well with the increased adhesion to HT-29 cells observed.

DnaK is another chaperone protein, the transcription of which is induced on exposure to environmental stresses (temperature, bile salts, etc.). We observed transcript levels for dnaK at levels that were discordant with those of other known chaperone proteins apart from groEL. It is interesting because as reported for groEL, dnaK is also regarded as having a potential for acting as a surface bound receptor in *Bifidobacterium animalis* and has been associated with increased adhesion to host tissues [53]. The experimental observations in this study appear to support a role for these two proteins in adhesion as exposure of bifidobacteria to milk oligosaccharides should not elicit a stress response. However in terms of intestinal colonisation, the ability to mount a response likely to facilitate adherence is advantageous to the bacteria and sustaining the level of dnaK and groEL transcript will obviously be of benefit under these conditions.

Although the sortase gene was not detected as being up-regulated by the microarray experiment, we did detect up-regulation by qPCR. The function of sortase enzymes in the anchoring of surface proteins is vital to their correct presentation on the external surface of bacterial cells. It is thought that the anchoring of sortase-dependent proteins may in some circumstances be limited by the availability of the sortase enzyme and therefore transcription of the sortase at higher levels would increase the anchoring of sortase-dependent proteins. Sortases are not essential for cell growth, as sortase deletion mutants are viable, however, adhesion of *Lactobacillus salivarius* to epithelial cells is reduced in sortase-negative mutants [54], [55].

The tight adherence locus (*tad*) is homologous in members of *Actinobacteria* [56] and is responsible for the formation and assembly of type IVB pili. These pili bind to carbohydrate moieties present in glycoprotein and glycolipid receptors and are thought to mediate the initial encounter with host structures [57]. TadE transcription, as measured using the microarrays, was not significantly increased in response to oligosaccharide treatment, however because this locus has been reported elsewhere to be an important mediator of colonisation in *B. breve* [21] we decided to examine the expression of the tadE gene by qPCR, where it was found to be transcribed at a higher level. This increased expression of tadE and its role in the assembly of proteinaceous surface appendages could potentially contribute to the enhanced adhesive response of *B. longum* subsp. *infantis* ATCC 15697.

It is not clear why this discrepancy between the array results and the qPCR results occurred for those genes, however, differences between transcriptional data obtained from microarray and qPCR experiments are not unknown and may arise in this instance from the fact that the microarrays used directly-labelled mRNA while the qPCR analysis was done on cDNA. In addition, the selection process for deciding if a particular locus is significant in the microarrays was a combination of fold-change and statistical significance and this process leads to genes that narrowly exceed the statistical cut-offs being disregarded.

A glycosyl hydrolase active against the GlcNAcβ1-3 linkages found in lacto-N-hexaose, an oligosaccharide abundant in human milk [6], was the only example of an up-regulated gene that was specifically related to sugar metabolism. In this study, this gene was only substantially differentially expressed in response to the mixture of 3′ and 6′sialyllactose. In addition to the glycosyl hydrolase, a sugar-binding ABC transporter component was also up-regulated (blon_2061). Of interest regarding this gene is that there are 6 similar binding proteins present in the genome of *B. longum* subsp. *infantis* 15697 yet blon_2061 is the only one up-regulated, indicating that the induction of this gene is treatment-specific and not a component of a broad-spectrum global transcriptional response to oligosaccharide exposure. The general tendency was for genes involved in carbohydrate metabolism to be down-regulated.

Interestingly, the presence of lactose in experimental media at equal concentration during bacterial exposure resulted in the elimination of both, the adhesive *in vitro* response, as well as the associated up-regulation of the selected genes of interest determined through qPCR. Lactose is largely digested and removed in the small intestine, while HMOs are able to resist digestion and reach the colon [16], [58]. Accordingly, the current data demonstrate the potential function of HMOs to act as a site-specific adhesion-promoting factor in the large intestine.

Examination of the down-regulated genes revealed the interesting result that the HMO-utilisation cluster described previously by Sela *et al.* [13] was generally down-regulated following all three oligosaccharide treatments. As this cluster of thirty contiguous genes encodes sialidases, fucosidases, β-galactosidases, solute-binding proteins, and ABC transport permeases [13], [59], it is likely most effective in the degradation of complex milk oligosaccharides and the presence of the relatively simple structures of 3′sialyllactose and 6′sialyllactose may well act to suppress the transcription of those genes involved in complex oligosaccharide hydrolysis. Furthermore, digestion and growth upon HMOs as a sole carbon source is typically observed between 20–48 hours following inoculation [60]. A second cluster of genes reported to be involved in metabolism of lacto-N-biose and galacto-N-biose (LNB/GNB metabolic pathway; blon_2171-blon_2177) [59] was also slightly down-regulated and again this down-regulation may be a consequence of the availability of less complex oligosaccharides. These findings relating to suppressed carbohydrate metabolism are supported by the HPLC results indicating that significant oligosaccharide metabolism was absent during the oligosaccharide exposure period of three hours.

This study has confirmed a substantial increase in the adhesive potential of *B. longum* subsp. *infantis* ATCC 15697 to HT-29 cells following certain oligosaccharide treatments and this correlated extremely well with the transcriptional response. The transcriptional response itself was composed of two distinct components in which there was a significant up-regulation of a number of genes likely to enhance adhesion to epithelial cells with an additional down-regulatory effect on the transcription of a group of genes likely to be involved in the metabolism of more complex oligosaccharides. The magnitude of the transcriptional response (and the enhanced adhesion phenotype) was greater during co-incubation with two oligosaccharides rather than either alone. Exposure of *B. longum* subsp. *infantis* ATCC 15697 to 6′sialyllactose at a concentration of 1 mg/mL for a duration of 3 hours results in a substantial increase in adhesion to the HT-29 cells compared to the non-oligosaccharide exposed control. While increasing exposure time or concentration did not produce a concentration-dependent effect, decreasing the oligosaccharide concentration to 0.5 mg/ml or below resulted in levels of adhesion to HT-29 cells that did not differ from the control samples. This would suggest that while relatively low concentrations of oligosaccharides are effective in promoting adhesion, there is a threshold value below which they have no effect. This would suggest that *B. longum* subsp. *infantis* ATCC 15697 possesses a sensitive detection mechanism for the presence of oligosaccharides that is active in the micromolar range and which responds to oligosaccharide concentrations between 0.5 mg/ml and 1 mg/ml. The quite narrow response range suggests a sensor that is adapted to respond to a relatively low and stable level of oligosaccharides likely to be present in the intestinal environment, as levels of 3′sialyllactose and 6′sialyllactose in human colostrum are 0.30 and 0.37 mg/mL, respectively, following the first 3 days of lactation [61] and thought to be further concentrated and enriched in the large intestine and colon [37].

The current findings suggest that *B. longum* subsp. *infantis* ATCC 15697 is capable of sensing subtle environmental signals such as oligosaccharides and mounting a proportional transcriptomic and physiological response. The ability to detect and respond to the presence of potentially beneficial adhesion-promoting molecules is of immense benefit to a commensal species exposed to the constantly changing gut environment. The results obtained from this study provide a new insight into the mechanisms by which commensal bacteria adapt to their residency in the intestinal tract and the role of ingested oligosaccharides in eliciting an adaptive response. Of particular interest is the identification of a number of genes for potential colonisation factors that are very similar to known virulence factors employed by pathogenic bacteria. The expression of these genes may give beneficial commensals such as *Bifidobacterium* the opportunity to compete with pathogenic bacteria for specific intestinal niches thereby reducing colonisation by pathogens. The induction of these responses by milk oligosaccharides provides a molecular-level explanation to aid in the identification and characterisation of the protective effects of milk oligosaccharides previously observed in other *in vivo* studies.

## Supporting Information

Figure S1 **Screening oligosaccharides for their ability to influence adhesion of** ***B. longum*** **subsp.** ***infantis*** **ATCC 15697 to Caco-2 monolayers.** Abbreviations: P95 - Beneo Orafti P95; 3′SL - 3′sialyllactose; 6′SL –6′sialyllactose; Lac - lactose. Non-supplemented tissue culture media was used as control. Results are expressed as fold-change relative to control percent adhesion with error bars representing standard deviation. ^a^denotes significant difference in relation to control; ^b^denotes significant difference in relation to P95 and LAC/6′SL groups; p = 0.0027.
(TIF)

Figure S2 **Boxplot analysis of the pool of genes up-regulated by the mixture of 3′- and 6′-sialyllactose.** Common = those genes upregulated by all treatments, [3′SL] & [3′+6′SL] = genes also upregulated by 3′SL; [6′SL] & [3′+6′SL] = genes also up-regulated by 6′SL alone; [3′+6′SL] = genes only up-regulated by the 3′- and 6′-sialyllactose mixture.
(TIF)

Figure S3 **Transcription levels (fold-change) for the cluster of genes for LNB/GNB metabolism.** 3′sialyllactose (white shading); 6′sialyllactose (black and white diagonal shading); 3′- and 6′-sialyllactose mixture (black shading).
(TIF)

Figure S4 **Transcription (fold-change) of the genes in the HMO-utilisation cluster (blon_2331-blon_2361) following exposure to the three oligosaccharide treatments.** 3′sialyllactose (white shading); 6′sialyllactose (black and white diagonal shading); 3′- and 6′-sialyllactose mixture (black shading).
(TIF)

Table S1 **Selected genes, primers, and probes for qPCR.**
(DOC)

Table S2 **List of genes differentially regulated by 3′sialyllactose treatment.**
(DOC)

Table S3 **List of genes differentially regulated by 6′sialyllactose treatment.**
(DOC)

Table S4 **List of genes differentially regulated by treatment with a mixture of 3′- and 6′-sialyllactose.**
(DOC)

Table S5 **List of common genes up-regulated by 6′sialyllactose and the mixture of 3′-and 6′-sialyllactose.**
(DOC)
