# Supplementary figures and images for: Exposure of Bifidobacterium longum subsp. infantis to Milk Oligosaccharides Increases Adhesion to Epithelial Cells and Induces a Substantial Transcriptional Response
Source: PLoS One. 2013 Jun 21;8(6):e67224. doi: 10.1371/journal.pone.0067224 (PMC3689703; doi:10.1371/journal.pone.0067224)

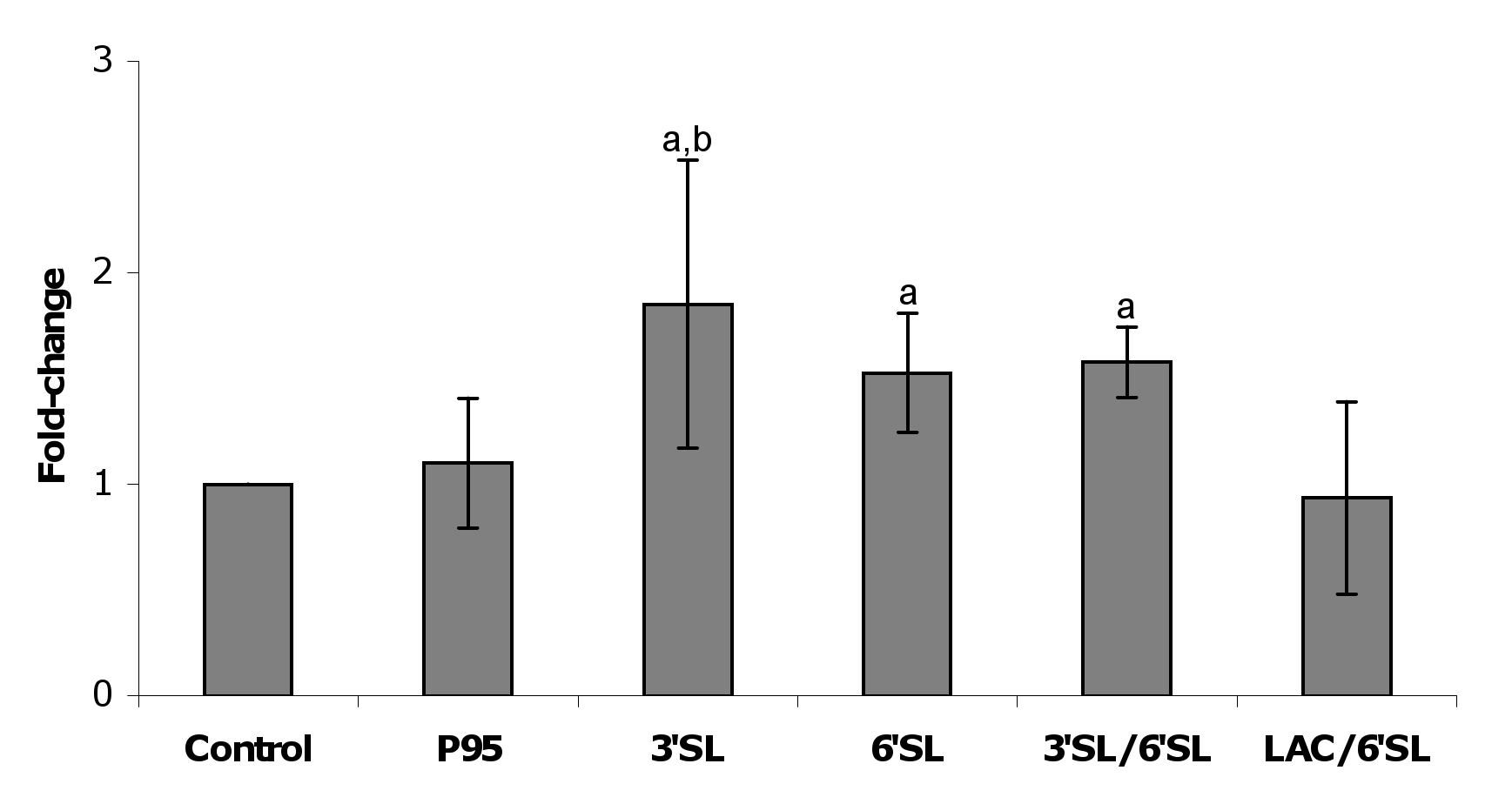

Supplement: Figure S1 — Screening oligosaccharides for their ability to influence adhesion of B. longum subsp. infantis ATCC 15697 to Caco-2 monolayers. Abbreviations: P95 - Beneo Orafti P95; 3′SL - 3′sialyllactose; 6′SL –6′sialyllactose; Lac - lactose. Non-supplemented tissue culture media was used as control. Results are expressed as fold-change relative to control percent adhesion with error bars representing standard deviation. adenotes significant difference in relation to control; bdenotes significant difference in relation to P95 and LAC/6′SL groups; p = 0.0027. (TIF) [file pone.0067224.s001.tif]

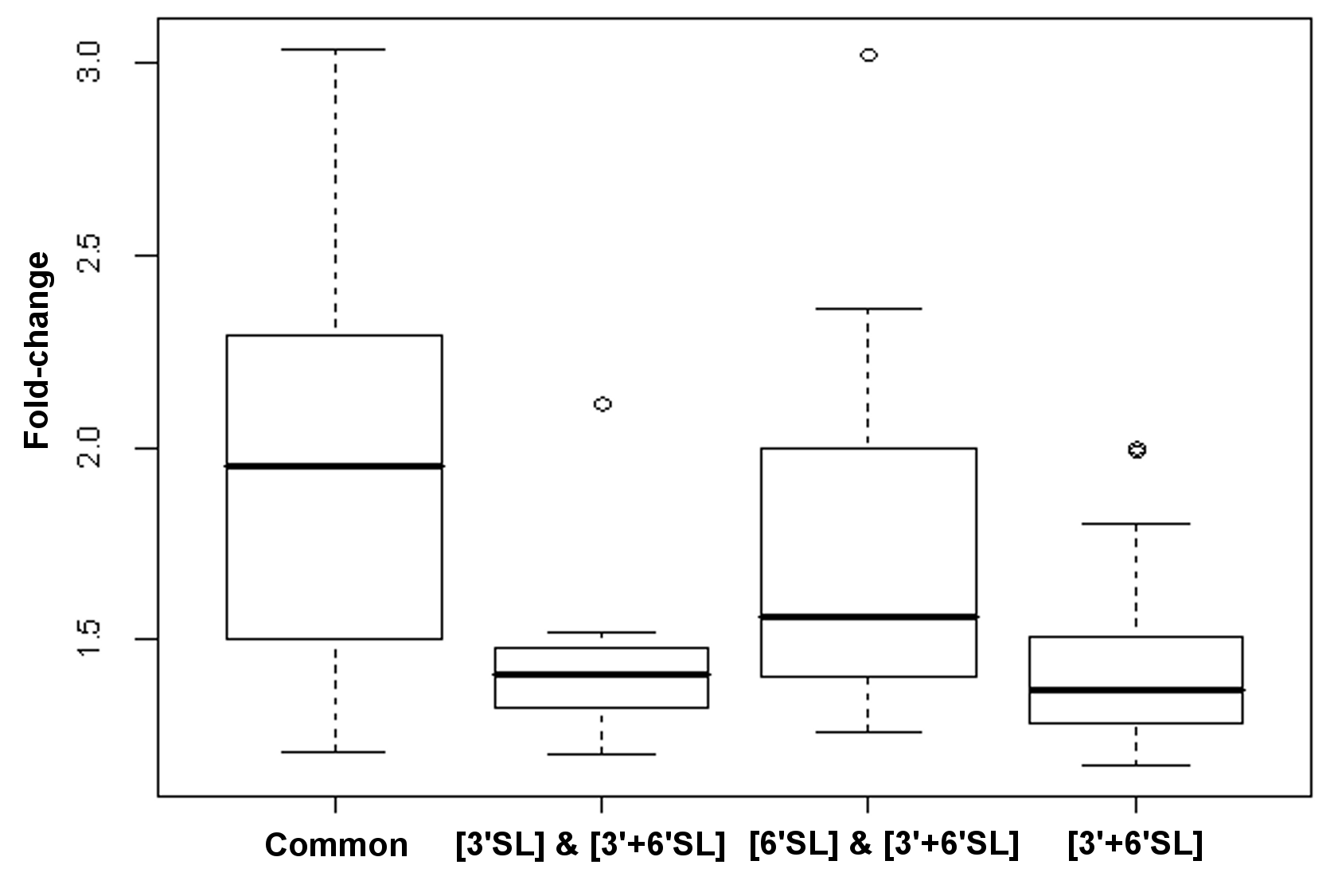

Supplement: Figure S2 — Boxplot analysis of the pool of genes up-regulated by the mixture of 3′- and 6′-sialyllactose. Common = those genes upregulated by all treatments, [3′SL] & [3′+6′SL] = genes also upregulated by 3′SL; [6′SL] & [3′+6′SL] = genes also up-regulated by 6′SL alone; [3′+6′SL] = genes only up-regulated by the 3′- and 6′-sialyllactose mixture. (TIF) [file pone.0067224.s002.tif]
