# Supplementary material for: Exposure of Bifidobacterium longum subsp. infantis to Milk Oligosaccharides Increases Adhesion to Epithelial Cells and Induces a Substantial Transcriptional Response
Source: PLoS One. 2013 Jun 21;8(6):e67224. doi: 10.1371/journal.pone.0067224 (PMC3689703; doi:10.1371/journal.pone.0067224)
Supplement: Table S1 — Selected genes, primers, and probes for qPCR. (DOC) [file pone.0067224.s005.doc]

Table S1. Selected genes, primers, and probes for qPCR.

| **Gene / Putative function** | **Primer (5’ – 3’)** | **Roche Probe #** |
| --- | --- | --- |
| Blon_1971 - High-affinity zinc ABC transporter | F: gtacgagcattcccacgac  R: acaggtgcgggttcgtag | 9 |
| Blon_0029 - Ferritin | F: tcctgcagaaccgactgag  R: gttccagtgtgcgtgcttc | 15 |
| Blon_0392 - Cation efflux protein | F: ggtcatctacgagatcctgacc  R: tggaacgccggtaatacg | 41 |
| Blon_0993 - Magnesium-translocating P-type ATPase | F: gtgaaaccggatcgtgatg  R: tccggtctggaagtagctgt | 65 |
| Blon_1687 - TfoX, C-terminal domain protein | F: ggtattttagatgaagacgatttgaa  R: tttgctgtattttcaaccatgc | 68 |
| Blon_1688 - Transcription activator, effector binding | F: cgctggaactggcaaaaa  R: caaccatgttccgaagatatactg | 4 |
| Blon_1990 - Glycine dehydrogenase | F: cgtatcatcgtccgcagtc  R: ccggatacgccgtctgt | 76 |
| Blon_0459 - Glycoside hydrolase, family 20 | F: ccgacaagctctgcctgta  R: gcgaaggtgtgttcgatgt | 78 |
| Blon_2061 – Extracellular solute binding protein | F: aggtttccggcactcacat  R: gcatctggagtcccagctt | 20 |
| Adhesion-related targets |  |  |
| Blon_0141 - Chaperonin protein DnaK | F: gcaggcgaagaaggaactc  R: gccaggtactgcatggaaat | 10 |
| Blon_0156 - TadE family protein | F: agactgccgccagacctat  R: cgtcctattgccagtatcagc | 61 |
| Blon_0162 - Sortase | F: cagcccatcttgggtgaa  R: ctggcttgggatcctcct | 70 |
| Blon_0694 - GroEL | F: gatgaggaagcccgtcag  R: tgaccttgacggtgttgg | 67 |
| Endogenous controls |  |  |
| Blon_0393 - Cysteinyl-tRNA synthetase | F: gcgatggcttcgacattc  R: ccatctcgttttcgtggtg | 70 |
| Blon_R0085 – 16S rRNA | F: acgggtgagtaatgcgtga  R: acccgtttccaggagctatt | 43 |
