# Supplementary material for: Exposure of Bifidobacterium longum subsp. infantis to Milk Oligosaccharides Increases Adhesion to Epithelial Cells and Induces a Substantial Transcriptional Response
Source: PLoS One. 2013 Jun 21;8(6):e67224. doi: 10.1371/journal.pone.0067224 (PMC3689703; doi:10.1371/journal.pone.0067224)
Supplement: Table S2 — List of genes differentially regulated by 3′sialyllactose treatment. (DOC) [file pone.0067224.s006.doc]

Table S2: List of genes differentially regulated by 3'sialyllactose treatment.

| **Gene Name** | **Description** | **P-Value** | **Fold Change** |
| --- | --- | --- | --- |
| Blon_0004 | DNA replication and repair protein RecF | 0.045 | 1.28 |
| Blon_0029 | Ferritin, Dps family protein | 0.000 | 1.67 |
| Blon_0036 | FAD-dependent pyridine nucleotide-disulphide oxidoreductase | 0.017 | 1.31 |
| Blon_0121 | hypothetical protein | 0.019 | 0.75 |
| Blon_0122 | hypothetical protein | 0.027 | 0.75 |
| Blon_0125 | Camphor resistance CrcB protein | 0.020 | 0.79 |
| Blon_0142 | GrpE protein | 0.045 | 1.24 |
| Blon_0221 | conserved hypothetical protein | 0.033 | 0.80 |
| Blon_0225 | (Protein-PII) uridylyltransferase | 0.015 | 0.76 |
| Blon_0264 | FAD dependent oxidoreductase | 0.025 | 1.35 |
| Blon_0335 | putative transcriptional regulator, MerR family | 0.041 | 0.84 |
| Blon_0336 | major facilitator superfamily MFS_1 | 0.050 | 0.75 |
| Blon_0392 | cation efflux protein | 0.001 | 1.66 |
| Blon_0460 | binding-protein-dependent transport systems inner membrane component | 0.037 | 1.32 |
| Blon_0505 | hypothetical protein | 0.020 | 0.80 |
| Blon_0612 | anaerobic ribonucleoside-triphosphate reductase | 0.022 | 1.27 |
| Blon_0613 | anaerobic ribonucleoside-triphosphate reductase activating protein | 0.026 | 1.38 |
| Blon_0617 | glutamate--cysteine ligase, GCS2 | 0.022 | 1.32 |
| Blon_0644 | ROK family protein | 0.044 | 0.77 |
| Blon_0645 | N-acylglucosamine-6-phosphate 2-epimerase | 0.029 | 0.73 |
| Blon_0646 | glycosyl hydrolase, BNR repeat-containing protein | 0.028 | 1.21 |
| Blon_0697 | two component transcriptional regulator, winged helix family | 0.009 | 1.37 |
| Blon_0702 | ATPase AAA-2 domain protein | 0.015 | 1.28 |
| Blon_0758 | Glutaredoxin-like protein | 0.004 | 1.45 |
| Blon_0759 | ABC transporter related | 0.029 | 1.42 |
| Blon_0779 | 1-deoxy-D-xylulose 5-phosphate reductoisomerase | 0.024 | 1.45 |
| Blon_0790 | proteinase inhibitor I4, serpin | 0.047 | 0.80 |
| Blon_0862 | ABC transporter related | 0.039 | 1.26 |
| Blon_0884 | binding-protein-dependent transport systems inner membrane component | 0.024 | 0.76 |
| Blon_0885 | binding-protein-dependent transport systems inner membrane component | 0.020 | 0.80 |
| Blon_0892 | conserved hypothetical protein | 0.043 | 1.26 |
| Blon_0902 | initiation factor 3 | 0.005 | 1.65 |
| Blon_0947 | helix-turn-helix domain protein | 0.010 | 1.32 |
| Blon_0948 | hypothetical protein Blon_0948 | 0.021 | 1.30 |
| Blon_0992 | hypothetical protein Blon_0992 | 0.021 | 1.43 |
| Blon_1007 | pyridoxamine 5'-phosphate oxidase-related, FMN-binding | 0.018 | 1.24 |
| Blon_1157 | Holliday junction DNA helicase RuvA | 0.048 | 1.26 |
| Blon_1205 | hypothetical protein | 0.042 | 0.80 |
| Blon_1337 | hypothetical protein | 0.024 | 0.71 |
| Blon_1417 | ABC transporter related | 0.040 | 0.80 |
| Blon_1574 | branched-chain amino acid aminotransferase | 0.040 | 0.83 |
| Blon_1575 | ribosomal 5S rRNA E-loop binding protein Ctc/L25/TL5 | 0.048 | 0.80 |
| Blon_1640 | DEAD/DEAH box helicase domain protein | 0.004 | 1.57 |
| Blon_1672 | IstB domain protein ATP-binding protein | 0.039 | 1.22 |
| Blon_1687 | TfoX, C-terminal domain protein | 0.008 | 1.56 |
| Blon_1688 | transcription activator, effector binding | 0.001 | 1.78 |
| Blon_1776 | GreA/GreB family elongation factor | 0.023 | 1.27 |
| Blon_1786 | hypothetical protein | 0.049 | 1.26 |
| Blon_1850 | regulatory protein GntR, HTH | 0.048 | 1.30 |
| Blon_1926 | hypothetical protein | 0.014 | 0.66 |
| Blon_1950 | hypothetical protein | 0.010 | 1.26 |
| Blon_1951 | UMUC domain protein DNA-repair protein | 0.012 | 1.28 |
| Blon_1958 | amidophosphoribosyltransferase | 0.029 | 1.30 |
| Blon_2174 | conserved hypothetical protein | 0.024 | 0.72 |
| Blon_2175 | binding-protein-dependent transport systems inner membrane component | 0.028 | 0.74 |
| Blon_2176 | binding-protein-dependent transport systems inner membrane component | 0.006 | 0.68 |
| Blon_2191 | ribose 5-phosphate isomerase | 0.025 | 1.23 |
| Blon_2341 | protein of unknown function | 0.019 | 0.77 |
| Blon_2370 | glycerophosphoryl diester phosphodiesterase | 0.000 | 1.71 |
| Blon_2371 | Glutamate--tRNA ligase | 0.002 | 1.45 |
| Blon_2372 | ATPase AAA-2 domain protein | 0.000 | 1.70 |
| Blon_2379 | binding-protein-dependent transport systems inner membrane component | 0.007 | 0.65 |
| Blon_2380 | extracellular solute-binding protein, family 1 | 0.003 | 0.66 |
| Blon_2476 | glycosyl transferase, family 2 | 0.046 | 0.83 |
| dnaK | chaperone protein DnaK | 0.002 | 1.44 |
| groEL | chaperonin GroEL | 0.024 | 1.33 |
| hrcA | heat-inducible transcription repressor HrcA | 0.043 | 1.25 |
| pheT | phenylalanyl-tRNA synthetase, beta subunit | 0.049 | 1.36 |
| recA | recA protein | 0.045 | 1.19 |
| rnc | Ribonuclease III | 0.041 | 1.39 |
| rplD | ribosomal protein L4/L1e | 0.047 | 1.29 |
| rplT | ribosomal protein L20 | 0.048 | 1.23 |
| rplW | Ribosomal protein L25/L23 | 0.042 | 1.29 |
| rpmA | ribosomal protein L27 | 0.042 | 1.23 |
| rpmF | ribosomal protein L32 | 0.027 | 1.28 |
| rpmI | ribosomal protein L35 | 0.005 | 1.49 |
| ruvB | Holliday junction DNA helicase RuvB | 0.043 | 1.25 |
