# Supplementary material for: Exposure of Bifidobacterium longum subsp. infantis to Milk Oligosaccharides Increases Adhesion to Epithelial Cells and Induces a Substantial Transcriptional Response
Source: PLoS One. 2013 Jun 21;8(6):e67224. doi: 10.1371/journal.pone.0067224 (PMC3689703; doi:10.1371/journal.pone.0067224)
Supplement: Table S3 — List of genes differentially regulated by 6′sialyllactose treatment. (DOC) [file pone.0067224.s007.doc]

Table S3: List of genes differentially regulated by 6'sialyllactose treatment.

| **Gene Name** | **Description** | **P-Value** | **Fold Change** |
| --- | --- | --- | --- |
| Blon_0028 | conserved hypothetical protein | 0.0491 | 1.35 |
| Blon_0029 | Ferritin, Dps family protein | 0.0002 | 1.72 |
| Blon_0036 | FAD-dependent pyridine nucleotide-disulphide oxidoreductase | 0.0365 | 1.26 |
| Blon_0268 | glycoside hydrolase family 2, sugar binding | 0.0044 | 0.71 |
| Blon_0286 | lactoylglutathione lyase (LGUL) family protein, diverged | 0.0441 | 1.91 |
| Blon_0291 | conserved hypothetical protein | 0.0095 | 1.58 |
| Blon_0307 | ATP synthase F1, alpha subunit | 0.0147 | 0.79 |
| Blon_0332 | hypothetical protein | 0.0270 | 0.79 |
| Blon_0335 | putative transcriptional regulator, MerR family | 0.0102 | 0.78 |
| Blon_0392 | cation efflux protein | 0.0001 | 1.84 |
| Blon_0394 | glutamine amidotransferase class-I | 0.0102 | 1.61 |
| Blon_0450 | hypothetical protein | 0.0308 | 1.31 |
| Blon_0459 | glycoside hydrolase, family 20 | 0.0118 | 1.65 |
| Blon_0460 | binding-protein-dependent transport systems inner membrane component | 0.0191 | 1.38 |
| Blon_0505 | hypothetical protein | 0.0194 | 0.79 |
| Blon_0518 | hypothetical protein | 0.0197 | 0.74 |
| Blon_0536 | hypothetical protein | 0.0216 | 1.26 |
| Blon_0573 | ROK family protein | 0.0341 | 0.75 |
| Blon_0615 | Resolvase, N-terminal domain protein | 0.0260 | 1.24 |
| Blon_0616 | transposase, IS605 OrfB family | 0.0446 | 1.25 |
| Blon_0617 | glutamate--cysteine ligase, GCS2 | 0.0009 | 1.63 |
| Blon_0619 | DNA polymerase, beta domain protein region | 0.0014 | 1.59 |
| Blon_0620 | nucleotidyltransferase substrate binding protein, HI0074 family | 0.0085 | 1.49 |
| Blon_0621 | Glucan 1,3-beta-glucosidase | 0.0462 | 1.34 |
| Blon_0643 | conserved hypothetical protein | 0.0308 | 0.78 |
| Blon_0644 | ROK family protein | 0.0204 | 0.73 |
| Blon_0645 | N-acylglucosamine-6-phosphate 2-epimerase | 0.0344 | 0.74 |
| Blon_0748 | Cystathionine gamma-synthase | 0.0484 | 0.83 |
| Blon_0758 | Glutaredoxin-like protein | 0.0044 | 1.45 |
| Blon_0759 | ABC transporter related | 0.0463 | 1.37 |
| Blon_0789 | periplasmic binding protein/LacI transcriptional regulator | 0.0456 | 0.83 |
| Blon_0790 | proteinase inhibitor I4, serpin | 0.0330 | 0.78 |
| Blon_0852 | UDP-N-acetylmuramoylalanyl-D-glutamyl-2,6-diaminopimelate--D-alanyl-D-alanyl ligase | 0.0453 | 0.82 |
| Blon_0862 | ABC transporter related | 0.0141 | 1.34 |
| Blon_0863 | ABC-2 type transporter | 0.0319 | 1.23 |
| Blon_0864 | ISXoo15 transposase | 0.0343 | 1.24 |
| Blon_0865 | putative transcriptional regulator | 0.0028 | 1.52 |
| Blon_0884 | binding-protein-dependent transport systems inner membrane component | 0.0295 | 0.77 |
| Blon_0885 | binding-protein-dependent transport systems inner membrane component | 0.0020 | 0.72 |
| Blon_0902 | initiation factor 3 | 0.0083 | 1.59 |
| Blon_0947 | helix-turn-helix domain protein | 0.0067 | 1.35 |
| Blon_0948 | hypothetical protein | 0.0199 | 1.30 |
| Blon_0991 | conserved hypothetical protein | 0.0032 | 1.90 |
| Blon_0992 | hypothetical protein | 0.0004 | 1.98 |
| Blon_0993 | hypothetical protein | 0.0008 | 1.79 |
| Blon_0994 | transcriptional regulator, Fis family | 0.0299 | 1.24 |
| Blon_1037 | conserved hypothetical protein | 0.0485 | 1.41 |
| Blon_1205 | hypothetical protein Blon_1205 | 0.0234 | 0.78 |
| Blon_1315 | transposase, IS605 OrfB family | 0.0072 | 1.29 |
| Blon_1495 | conserved hypothetical protein | 0.0114 | 1.39 |
| Blon_1496 | helix-turn-helix domain protein | 0.0095 | 1.54 |
| Blon_1539 | hypothetical protein | 0.0392 | 1.26 |
| Blon_1540 | hypothetical protein | 0.0293 | 1.25 |
| Blon_1541 | hypothetical protein | 0.0186 | 1.41 |
| Blon_1542 | hypothetical protein | 0.0218 | 1.39 |
| Blon_1545 | Cpl-7 lysozyme, C-terminal domain protein | 0.0364 | 1.28 |
| Blon_1664 | GCN5-related N-acetyltransferase | 0.0463 | 1.41 |
| Blon_1687 | TfoX, C-terminal domain protein | 0.0006 | 1.93 |
| Blon_1688 | transcription activator, effector binding | 0.0000 | 2.35 |
| Blon_1689 | GTP-binding protein YchF | 0.0200 | 1.41 |
| Blon_1691 | proline iminopeptidase | 0.0064 | 1.31 |
| Blon_1692 | integral membrane sensor signal transduction histidine kinase | 0.0004 | 1.83 |
| Blon_1693 | two component transcriptional regulator, LuxR family | 0.0007 | 1.72 |
| Blon_1697 | Phosphomethylpyrimidine kinase type-1 | 0.0020 | 1.74 |
| Blon_1698 | protein of unknown function UPF0102 | 0.0006 | 1.82 |
| Blon_1700 | SMF family protein | 0.0422 | 1.26 |
| Blon_1713 | narrowly conserved hypothetical protein | 0.0063 | 0.74 |
| Blon_1714 | pyruvate formate-lyase activating enzyme | 0.0252 | 0.80 |
| Blon_1761 | 1,4-alpha-glucan branching enzyme | 0.0241 | 0.77 |
| Blon_1902 | conserved hypothetical protein | 0.0035 | 0.73 |
| Blon_1910 | conserved hypothetical protein | 0.0103 | 1.34 |
| Blon_1950 | hypothetical protein | 0.0313 | 1.20 |
| Blon_1951 | UMUC domain protein DNA-repair protein | 0.0332 | 1.22 |
| Blon_2061 | extracellular solute-binding protein, family 1 | 0.0254 | 1.29 |
| Blon_2064 | transcriptional regulator, DeoR family | 0.0334 | 1.24 |
| Blon_2081 | conserved hypothetical protein | 0.0199 | 1.29 |
| Blon_2082 | lipopolysaccharide biosynthesis | 0.0112 | 1.50 |
| Blon_2173 | aminoglycoside phosphotransferase | 0.0236 | 0.79 |
| Blon_2174 | conserved hypothetical protein | 0.0060 | 0.64 |
| Blon_2175 | binding-protein-dependent transport systems inner membrane component | 0.0033 | 0.64 |
| Blon_2176 | binding-protein-dependent transport systems inner membrane component | 0.0013 | 0.62 |
| Blon_2186 | narrowly conserved hypothetical protein | 0.0345 | 1.27 |
| Blon_2191 | ribose 5-phosphate isomerase | 0.0158 | 1.25 |
| Blon_2335 | conserved hypothetical protein | 0.0427 | 0.83 |
| Blon_2341 | protein of unknown function DUF624 | 0.0113 | 0.75 |
| Blon_2342 | binding-protein-dependent transport systems inner membrane component | 0.0086 | 0.76 |
| Blon_2348 | Exo-alpha-sialidase | 0.0355 | 0.78 |
| Blon_2349 | dihydrodipicolinate synthetase | 0.0487 | 0.83 |
| Blon_2370 | glycerophosphoryl diester phosphodiesterase | 0.0001 | 1.83 |
| Blon_2371 | Glutamate--tRNA ligase | 0.0001 | 1.75 |
| Blon_2372 | ATPase AAA-2 domain protein | 0.0000 | 1.88 |
| Blon_2379 | binding-protein-dependent transport systems inner membrane component | 0.0221 | 0.70 |
| Blon_2380 | extracellular solute-binding protein, family 1 | 0.0115 | 0.73 |
| dnaK | chaperone protein DnaK | 0.0005 | 1.58 |
| groEL | chaperonin GroEL | 0.0147 | 1.37 |
| recA | recA protein | 0.0135 | 1.26 |
| rpmI | ribosomal protein L35 | 0.0055 | 1.47 |
| thiG | thiazole biosynthesis family protein | 0.04100675 | 0.80 |
