# Supplementary material for: Exposure of Bifidobacterium longum subsp. infantis to Milk Oligosaccharides Increases Adhesion to Epithelial Cells and Induces a Substantial Transcriptional Response
Source: PLoS One. 2013 Jun 21;8(6):e67224. doi: 10.1371/journal.pone.0067224 (PMC3689703; doi:10.1371/journal.pone.0067224)
Supplement: Table S4 — List of genes differentially regulated by treatment with a mixture of 3′- and 6′-sialyllactose. (DOC) [file pone.0067224.s008.doc]

Table S4: List of genes differentially regulated by treatment with a mixture of 3'- and 6'-sialyllactose.

| **Gene Name** | **Description** | **P-Value** | **Fold Change** |
| --- | --- | --- | --- |
| aspA | Fumarate Lyase | 0.0025 | 1.52 |
| Blon_0019 | Metallophosphoesterase | 0.0205 | 1.60 |
| Blon_0022 | phosphonate ABC transporter, ATPase subunit | 0.0146 | 1.35 |
| Blon_0029 | Ferritin, Dps family protein | 0.0002 | 2.79 |
| Blon_0035 | alkyl hydroperoxide reductase/ Thiol specific antioxidant/ Mal allergen | 0.0010 | 1.49 |
| Blon_0036 | FAD-dependent pyridine nucleotide-disulphide oxidoreductase | 0.0001 | 1.78 |
| Blon_0096 | ThiJ/PfpI domain protein | 0.0140 | 1.54 |
| Blon_0119 | Uracil-DNA glycosylase superfamily | 0.0106 | 0.76 |
| Blon_0120 | fructose-bisphosphate aldolase, class II | 0.0058 | 0.73 |
| Blon_0121 | hypothetical protein | 0.0016 | 0.68 |
| Blon_0122 | hypothetical protein | 0.0041 | 0.66 |
| Blon_0123 | Adenylosuccinate synthase | 0.0168 | 0.73 |
| Blon_0124 | Cl- channel, voltage-gated family protein | 0.0015 | 0.71 |
| Blon_0125 | Camphor resistance CrcB protein | 0.0001 | 0.59 |
| Blon_0127 | regulatory protein, LacI | 0.0054 | 0.76 |
| Blon_0139 | 4-alpha-glucanotransferase | 0.0428 | 1.55 |
| Blon_0142 | GrpE protein | 0.0095 | 1.34 |
| Blon_0176 | regulatory protein, LacI | 0.0424 | 1.27 |
| Blon_0182 | conserved hypothetical protein | 0.0046 | 1.29 |
| Blon_0200 | protein of unknown function DUF214 | 0.0050 | 0.77 |
| Blon_0221 | conserved hypothetical protein | 0.0100 | 0.76 |
| Blon_0222 | signal recognition particle-docking protein FtsY | 0.0031 | 0.74 |
| Blon_0223 | ammonium transporter | 0.0284 | 0.80 |
| Blon_0224 | nitrogen regulatory protein P-II | 0.0231 | 0.83 |
| Blon_0225 | (Protein-PII) uridylyltransferase | 0.0006 | 0.64 |
| Blon_0255 | tRNA/rRNA methyltransferase (SpoU) | 0.0304 | 0.85 |
| Blon_0261 | GCN5-related N-acetyltransferase | 0.0490 | 0.83 |
| Blon_0266 | periplasmic binding protein/LacI transcriptional regulator | 0.0463 | 0.80 |
| Blon_0268 | glycoside hydrolase family 2, sugar binding | 0.0023 | 0.72 |
| Blon_0286 | lactoylglutathione lyase (LGUL) family protein, diverged | 0.0019 | 3.03 |
| Blon_0291 | conserved hypothetical protein | 0.0004 | 2.02 |
| Blon_0292 | helix-turn-helix domain protein | 0.0295 | 1.75 |
| Blon_0306 | ATP synthase F1, delta subunit | 0.0434 | 0.77 |
| Blon_0308 | ATP synthase F1, gamma subunitC | 0.0265 | 0.74 |
| Blon_0326 | MscS Mechanosensitive ion channel | 0.0069 | 1.46 |
| Blon_0328 | transcriptional regulator, TetR family | 0.0143 | 0.77 |
| Blon_0332 | hypothetical protein Blon_0332 | 0.0255 | 0.78 |
| Blon_0335 | putative transcriptional regulator, MerR family | 0.0056 | 0.77 |
| Blon_0336 | major facilitator superfamily MFS_1 | 0.0116 | 0.72 |
| Blon_0339 | DEAD/DEAH box helicase domain protein | 0.0180 | 0.68 |
| Blon_0341 | Binding-protein-dependent transport systems inner membrane component | 0.0263 | 0.65 |
| Blon_0342 | Binding-protein-dependent transport systems inner membrane component | 0.0326 | 0.72 |
| Blon_0349 | Putative methyltransferase | 0.0463 | 1.17 |
| Blon_0392 | Cation efflux protein | 0.0000 | 3.04 |
| Blon_0450 | Hypothetical protein | 0.0009 | 1.57 |
| Blon_0459 | Glycoside hydrolase, family 20 | 0.0029 | 2.36 |
| Blon_0460 | Binding-protein-dependent transport systems inner membrane component | 0.0013 | 1.81 |
| Blon_0488 | Hypothetical protein | 0.0435 | 1.55 |
| Blon_0505 | Hypothetical protein | 0.0103 | 0.79 |
| Blon_0517 | ABC transporter related | 0.0359 | 0.70 |
| Blon_0527 | Hypothetical protein | 0.0037 | 0.59 |
| Blon_0528 | Hypothetical protein | 0.0151 | 0.70 |
| Blon_0529 | Hypothetical protein | 0.0042 | 0.68 |
| Blon_0530 | Conserved hypothetical protein | 0.0045 | 0.68 |
| Blon_0531 | Transcription factor WhiB | 0.0256 | 0.73 |
| Blon_0536 | Hypothetical protein | 0.0456 | 1.46 |
| Blon_0552 | 2-amino-4-hydroxy-6-hydroxymethyldihydropteridine pyrophosphokinase | 0.0229 | 0.75 |
| Blon_0556 | Hypothetical protein | 0.0176 | 1.55 |
| Blon_0567 | 2,5-didehydrogluconate reductase | 0.0211 | 1.25 |
| Blon_0570 | Conserved hypothetical membrane protein possibly involved in transport | 0.0133 | 1.26 |
| Blon_0572 | Carbohydrate kinase, FGGY | 0.0195 | 1.30 |
| Blon_0573 | ROK family protein | 0.0407 | 0.78 |
| Blon_0577 | Extracellular ligand-binding receptor | 0.0010 | 0.54 |
| Blon_0580 | ABC transporter related | 0.0006 | 0.60 |
| Blon_0605 | Endonuclease/exonuclease/phosphatase | 0.0304 | 0.67 |
| Blon_0612 | Anaerobic ribonucleoside-triphosphate reductase | 0.0494 | 1.20 |
| Blon_0615 | Resolvase, N-terminal domain protein | 0.0205 | 1.26 |
| Blon_0617 | Glutamate--cysteine ligase, GCS2 | 0.0143 | 1.37 |
| Blon_0638 | Purine nucleosidase | 0.0223 | 0.74 |
| Blon_0642 | GntR domain protein | 0.0228 | 0.76 |
| Blon_0643 | Conserved hypothetical protein | 0.0017 | 0.61 |
| Blon_0644 | ROK family protein | 0.0082 | 0.61 |
| Blon_0645 | N-acylglucosamine-6-phosphate 2-epimerase | 0.0028 | 0.59 |
| Blon_0646 | Glycosyl hydrolase, BNR repeat-containing protein | 0.0001 | 1.48 |
| Blon_0647 | Extracellular solute-binding protein, family 5 | 0.0160 | 0.71 |
| Blon_0648 | Binding-protein-dependent transport systems inner membrane component | 0.0461 | 0.55 |
| Blon_0649 | Oligopeptide/dipeptide ABC transporter, ATPase subunit | 0.0047 | 0.61 |
| Blon_0650 | ABC transporter related | 0.0066 | 0.59 |
| Blon_0651 | Dihydrodipicolinate synthetase | 0.0229 | 0.62 |
| Blon_0667 | Mandelate racemase/muconate lactonizing enzyme, N-terminal domain protein | 0.0043 | 0.69 |
| Blon_0697 | Two component transcriptional regulator, winged helix family | 0.0174 | 1.34 |
| Blon_0699 | Putative cold-shock DNA-binding domain protein | 0.0416 | 1.27 |
| Blon_0701 | UspA domain protein | 0.0048 | 1.51 |
| Blon_0702 | ATPase AAA-2 domain protein | 0.0058 | 1.28 |
| Blon_0710 | Extracellular solute-binding protein, family 3 | 0.0164 | 1.23 |
| Blon_0732 | Glycoside hydrolase, family 20 | 0.0306 | 1.21 |
| Blon_0744 | Polar amino acid ABC transporter, inner membrane subunit | 0.0465 | 0.63 |
| Blon_0748 | Cystathionine gamma-synthase | 0.0189 | 0.79 |
| Blon_0758 | Glutaredoxin-like protein | 0.0007 | 1.99 |
| Blon_0759 | ABC transporter related | 0.0067 | 1.93 |
| Blon_0761 | Polar amino acid ABC transporter, inner membrane subunit | 0.0252 | 1.27 |
| Blon_0762 | Band 7 protein | 0.0220 | 1.32 |
| Blon_0772 | Conserved hypothetical protein | 0.0100 | 1.33 |
| Blon_0785 | Membrane lipoprotein lipid attachment site | 0.0034 | 0.72 |
| Blon_0789 | Periplasmic binding protein/LacI transcriptional regulator | 0.0016 | 0.72 |
| Blon_0790 | Proteinase inhibitor I4, serpin | 0.0035 | 0.68 |
| Blon_0799 | Putative TIM-barrel protein, nifR3 family | 0.0413 | 0.62 |
| Blon_0838 | Methyltransferase small | 0.0351 | 0.75 |
| Blon_0840 | L-lactate dehydrogenase | 0.0226 | 1.72 |
| Blon_0841 | Cation diffusion facilitator family transporter | 0.0301 | 0.65 |
| Blon_0850 | Peptidoglycan glycosyltransferase | 0.0075 | 0.72 |
| Blon_0851 | Conserved hypothetical protein | 0.0007 | 0.68 |
| Blon_0852 | UDP-N-acetylmuramoylalanyl-D-glutamyl-2,6-diaminopimelate--D-alanyl-D-alanyl ligase | 0.0066 | 0.77 |
| Blon_0865 | Putative transcriptional regulator | 0.0012 | 1.89 |
| Blon_0879 | ROK family protein | 0.0324 | 1.34 |
| Blon_0884 | Binding-protein-dependent transport systems inner membrane component | 0.0484 | 0.68 |
| Blon_0885 | Binding-protein-dependent transport systems inner membrane component | 0.0001 | 0.67 |
| Blon_0902 | Initiation factor 3 | 0.0121 | 1.43 |
| Blon_0916 | Hypothetical protein | 0.0212 | 1.80 |
| Blon_0917 | hypothetical protein | 0.0068 | 1.36 |
| Blon_0918 | Prephenate dehydratase | 0.0106 | 1.55 |
| Blon_0938 | protein of unknown function UPF0005 | 0.0029 | 1.53 |
| Blon_0947 | helix-turn-helix domain protein | 0.0002 | 1.54 |
| Blon_0948 | hypothetical protein | 0.0025 | 1.50 |
| Blon_0951 | sigma 54 modulation protein/ribosomal protein S30EA | 0.0166 | 1.41 |
| Blon_0960 | RNA polymerase, sigma 70 subunit, RpoD family | 0.0213 | 0.76 |
| Blon_0971 | (p)ppGpp synthetase I, SpoT/RelA | 0.0101 | 0.73 |
| Blon_0991 | conserved hypothetical protein | 0.0039 | 2.17 |
| Blon_0992 | hypothetical protein | 0.0021 | 2.47 |
| Blon_0993 | hypothetical protein | 0.0047 | 2.05 |
| Blon_0994 | transcriptional regulator, Fis family | 0.0098 | 1.48 |
| Blon_0995 | ABC transporter related | 0.0377 | 1.49 |
| Blon_0999 | ABC transporter related | 0.0198 | 1.28 |
| Blon_1002 | hypothetical protein | 0.0418 | 1.45 |
| Blon_1007 | pyridoxamine 5'-phosphate oxidase-related, FMN-binding | 0.0020 | 2.11 |
| Blon_1023 | hypothetical protein | 0.0084 | 1.38 |
| Blon_1028 | hypothetical protein | 0.0468 | 1.52 |
| Blon_1037 | conserved hypothetical protein | 0.0034 | 1.85 |
| Blon_1038 | hypothetical protein | 0.0108 | 0.63 |
| Blon_1054 | AMP-dependent synthetase and ligase | 0.0177 | 1.20 |
| Blon_1063 | imidazoleglycerol phosphate synthase, cyclase subunit | 0.0331 | 0.63 |
| Blon_1092 | aminotransferase, class I and II | 0.0259 | 0.74 |
| Blon_1150 | fructosamine kinase | 0.0305 | 1.27 |
| Blon_1154 | histidine triad (HIT) protein | 0.0110 | 0.71 |
| Blon_1157 | Holliday junction DNA helicase RuvA | 0.0195 | 1.32 |
| Blon_1166 | pseudouridine synthase | 0.0289 | 0.82 |
| Blon_1225 | Resolvase, N-terminal domain protein | 0.0424 | 1.24 |
| Blon_1338 | putative transcriptional regulator, XRE family | 0.0385 | 1.22 |
| Blon_1387 | conserved hypothetical protein | 0.0300 | 1.29 |
| Blon_1426 | hypothetical protein | 0.0069 | 0.71 |
| Blon_1455 | 5-methyltetrahydropteroyltriglutamate--homocysteine S-methyltransferase | 0.0280 | 0.74 |
| Blon_1492 | hypothetical protein Blon_1492 | 0.0360 | 1.20 |
| Blon_1494 | hypothetical protein | 0.0357 | 1.32 |
| Blon_1495 | conserved hypothetical protein | 0.0153 | 1.40 |
| Blon_1496 | helix-turn-helix domain protein | 0.0170 | 1.47 |
| Blon_1541 | hypothetical protein | 0.0288 | 1.33 |
| Blon_1574 | branched-chain amino acid aminotransferase | 0.0009 | 0.67 |
| Blon_1613 | ABC transporter related | 0.0216 | 0.70 |
| Blon_1615 | conserved hypothetical protein | 0.0144 | 0.68 |
| Blon_1634 | protein of unknown function DUF47 | 0.0290 | 1.44 |
| Blon_1664 | GCN5-related N-acetyltransferase | 0.0080 | 1.60 |
| Blon_1679 | transcriptional modulator of MazE/toxin, MazF | 0.0065 | 1.39 |
| Blon_1687 | TfoX, C-terminal domain protein | 0.0014 | 2.26 |
| Blon_1688 | transcription activator, effector binding | 0.0001 | 2.88 |
| Blon_1693 | two component transcriptional regulator, LuxR family | 0.0349 | 1.32 |
| Blon_1698 | protein of unknown function UPF0102 | 0.0096 | 1.38 |
| Blon_1708 | Endopeptidase Clp | 0.0166 | 0.73 |
| Blon_1709 | hypothetical protein | 0.0033 | 0.68 |
| Blon_1710 | Cl- channel, voltage-gated family protein | 0.0335 | 0.78 |
| Blon_1712 | 3'-5' exonuclease | 0.0281 | 0.65 |
| Blon_1713 | narrowly conserved hypothetical protein | 0.0054 | 0.65 |
| Blon_1745 | Pyruvate kinase | 0.0323 | 1.34 |
| Blon_1756 | periplasmic solute binding protein | 0.0037 | 1.28 |
| Blon_1761 | 1,4-alpha-glucan branching enzyme | 0.0107 | 0.74 |
| Blon_1771 | cell envelope-related transcriptional attenuator | 0.0317 | 0.67 |
| Blon_1773 | transcription factor WhiB | 0.0260 | 1.38 |
| Blon_1774 | signal transduction histidine kinase | 0.0329 | 0.81 |
| Blon_1776 | GreA/GreB family elongation factor | 0.0003 | 1.52 |
| Blon_1777 | peptidylprolyl isomerase, FKBP-type | 0.0022 | 1.40 |
| Blon_1780 | phage integrase family protein | 0.0185 | 0.77 |
| Blon_1786 | hypothetical protein | 0.0146 | 1.31 |
| Blon_1839 | Aminoacyl-tRNA hydrolase | 0.0161 | 0.69 |
| Blon_1850 | regulatory protein GntR, HTH | 0.0067 | 1.41 |
| Blon_1855 | regulatory protein GntR, HTH | 0.0239 | 1.33 |
| Blon_1856 | two component transcriptional regulator, LuxR family | 0.0469 | 1.32 |
| Blon_1857 | Signal transduction histidine kinase-like protein | 0.0106 | 1.30 |
| Blon_1873 | thiamine biosynthesis protein ThiS | 0.0488 | 0.84 |
| Blon_1876 | arginine repressor, ArgR | 0.0103 | 0.75 |
| Blon_1879 | acetylglutamate kinase | 0.0181 | 0.62 |
| Blon_1901 | ABC transporter related | 0.0117 | 0.74 |
| Blon_1902 | conserved hypothetical protein | 0.0089 | 0.73 |
| Blon_1950 | hypothetical protein | 0.0017 | 1.35 |
| Blon_1951 | UMUC domain protein DNA-repair protein | 0.0012 | 1.41 |
| Blon_1968 | ribosomal protein L31 | 0.0095 | 1.50 |
| Blon_1969 | ribosomal protein L36 | 0.0345 | 1.47 |
| Blon_1971 | putative high-affinity zinc ABC transporter | 0.0139 | 2.01 |
| Blon_1990 | hypothetical protein | 0.0152 | 1.99 |
| Blon_2022 | extracellular solute-binding protein, family 3 | 0.0115 | 1.35 |
| Blon_2048 | DNA-directed RNA polymerase, beta' subunit | 0.0441 | 0.79 |
| Blon_2061 | extracellular solute-binding protein, family 1 | 0.0000 | 1.98 |
| Blon_2062 | galactokinase | 0.0079 | 1.58 |
| Blon_2063 | galactose-1-phosphate uridylyltransferase | 0.0078 | 1.52 |
| Blon_2064 | transcriptional regulator, DeoR family | 0.0047 | 1.55 |
| Blon_2082 | lipopolysaccharide biosynthesis | 0.0175 | 1.41 |
| Blon_2086 | transcriptional regulator, Fis family | 0.0202 | 1.39 |
| Blon_2098 | IS3 family transposase | 0.0358 | 0.75 |
| Blon_2138 | protein of unknown function | 0.0072 | 1.36 |
| Blon_2171 | UDP-glucose 4-epimerase | 0.0028 | 1.47 |
| Blon_2172 | UDP-glucose--hexose-1-phosphate uridylyltransferase | 0.0313 | 0.64 |
| Blon_2173 | aminoglycoside phosphotransferase | 0.0214 | 0.77 |
| Blon_2174 | conserved hypothetical protein | 0.0028 | 0.57 |
| Blon_2175 | binding-protein-dependent transport systems inner membrane component | 0.0146 | 0.70 |
| Blon_2176 | binding-protein-dependent transport systems inner membrane component | 0.0019 | 0.66 |
| Blon_2177 | extracellular solute-binding protein, family 1 | 0.0307 | 0.78 |
| Blon_2187 | transcriptional regulator, BadM/Rrf2 family | 0.0077 | 1.45 |
| Blon_2188 | pyridine nucleotide-disulphide oxidoreductase dimerisation region | 0.0452 | 1.20 |
| Blon_2191 | ribose 5-phosphate isomerase | 0.0014 | 1.58 |
| Blon_2264 | large hypothetical protein | 0.0126 | 0.74 |
| Blon_2273 | ABC transporter related | 0.0479 | 0.77 |
| Blon_2287 | hypothetical protein | 0.0210 | 1.23 |
| Blon_2326 | regulatory protein, IclR | 0.0382 | 1.27 |
| Blon_2335 | conserved hypothetical protein | 0.0029 | 0.72 |
| Blon_2336 | alpha-1,3/4-fucosidase, putative | 0.0151 | 0.69 |
| Blon_2337 | RbsD or FucU transport | 0.0180 | 0.65 |
| Blon_2341 | protein of unknown function | 0.0017 | 0.58 |
| Blon_2342 | binding-protein-dependent transport systems inner membrane component | 0.0003 | 0.67 |
| Blon_2343 | binding-protein-dependent transport systems inner membrane component | 0.0311 | 0.72 |
| Blon_2348 | Exo-alpha-sialidase | 0.0247 | 0.68 |
| Blon_2349 | dihydrodipicolinate synthetase | 0.0125 | 0.78 |
| Blon_2355 | glycoside hydrolase, family 20 | 0.0449 | 0.63 |
| Blon_2370 | glycerophosphoryl diester phosphodiesterase | 0.0000 | 2.09 |
| Blon_2371 | Glutamate--tRNA ligase | 0.0000 | 2.29 |
| Blon_2372 | ATPase AAA-2 domain protein | 0.0000 | 2.45 |
| Blon_2379 | binding-protein-dependent transport systems inner membrane component | 0.0022 | 0.54 |
| Blon_2380 | extracellular solute-binding protein, family 1 | 0.0007 | 0.65 |
| Blon_2386 | ABC transporter related | 0.0237 | 0.80 |
| Blon_2387 | ABC-2 type transporter | 0.0451 | 0.66 |
| Blon_2397 | K+ potassium transporter | 0.0411 | 0.71 |
| Blon_2444 | extracellular solute-binding protein, family 1 | 0.0405 | 1.61 |
| Blon_2474 | narrowly conserved hypothetical protein | 0.0044 | 0.69 |
| Blon_2475 | ABC transporter related | 0.0359 | 0.79 |
| Blon_2476 | glycosyl transferase, family 2 | 0.0365 | 0.84 |
| Blon_2478 | ribonucleoside-diphosphate reductase, alpha subunit | 0.0149 | 0.57 |
| Blon_2492 | parB-like partition protein | 0.0169 | 0.78 |
| Blon_2496 | 60 kDa inner membrane insertion protein | 0.0192 | 0.59 |
| Blon_2497 | protein of unknown function DUF37 | 0.0388 | 0.56 |
| Blon_2498 | ribonuclease P protein component | 0.0228 | 0.72 |
| dnaK | chaperone protein DnaK | 0.0001 | 1.97 |
| groEL | chaperonin GroEL | 0.0002 | 2.11 |
| groES | chaperonin Cpn10 | 0.0029 | 1.34 |
| hisS | Histidine--tRNA ligase | 0.0282 | 0.70 |
| hrcA | heat-inducible transcription repressor HrcA | 0.0018 | 1.52 |
| ileS | isoleucyl-tRNA synthetase | 0.0135 | 0.75 |
| nagB | glucosamine-6-phosphate isomerase | 0.0006 | 1.45 |
| nrdI | NrdI protein | 0.0146 | 0.60 |
| pgi | Glucose-6-phosphate isomerase | 0.0083 | 1.36 |
| prfA | peptide chain release factor 1 | 0.0069 | 0.71 |
| recA | recA protein | 0.0267 | 1.21 |
| rplQ | ribosomal protein L17 | 0.0160 | 0.73 |
| rpmA | ribosomal protein L27 | 0.0043 | 1.42 |
| rpmD | ribosomal protein L30 | 0.0314 | 0.65 |
| rpmF | ribosomal protein L32 | 0.0025 | 1.45 |
| rpoB | DNA-directed RNA polymerase, beta subunit | 0.0259 | 0.77 |
| rpsH | ribosomal protein S8 | 0.0205 | 0.65 |
| rpsM | ribosomal protein S13 | 0.0082 | 0.79 |
| smpB | SsrA-binding protein | 0.0027 | 1.51 |
| thiG | thiazole biosynthesis family protein | 0.0027 | 0.70 |
| thrS | threonyl-tRNA synthetase | 0.0328 | 0.63 |
| tsf | translation elongation factor Ts | 0.0162 | 0.81 |
