# Supplementary material for: Exposure of Bifidobacterium longum subsp. infantis to Milk Oligosaccharides Increases Adhesion to Epithelial Cells and Induces a Substantial Transcriptional Response
Source: PLoS One. 2013 Jun 21;8(6):e67224. doi: 10.1371/journal.pone.0067224 (PMC3689703; doi:10.1371/journal.pone.0067224)
Supplement: Table S5 — List of common genes up-regulated by 6′sialyllactose and the mixture of 3′-and 6′-sialyllactose. (DOC) [file pone.0067224.s009.doc]

Table S5: List of common genes up-regulated by 6'sialyllactose and the mixture of 3'-and 6'-sialyllactose.

| **Locus Tag** | **Gene Description** |
| --- | --- |
| Blon_0029 | Ferritin, Dps family protein |
| Blon_0036 | FAD-dependent pyridine nucleotide-disulphide oxidoreductase |
| Blon_0286 | lactoylglutathione lyase (LGUL) family protein, diverged |
| Blon_0291 | conserved hypothetical protein |
| Blon_0392 | Cation efflux protein |
| Blon_0450 | Hypothetical protein |
| Blon_0459 | Glycoside hydrolase, family 20 |
| Blon_0460 | Binding-protein-dependent transport systems inner membrane component |
| Blon_0536 | Hypothetical protein |
| Blon_0615 | Resolvase, N-terminal domain protein |
| Blon_0617 | Glutamate--cysteine ligase, GCS2 |
| Blon_0758 | Glutaredoxin-like protein |
| Blon_0759 | ABC transporter related |
| Blon_0865 | Putative transcriptional regulator |
| Blon_0902 | Initiation factor 3 |
| Blon_0947 | helix-turn-helix domain protein |
| Blon_0948 | hypothetical protein |
| Blon_0991 | conserved hypothetical protein |
| Blon_0992 | hypothetical protein |
| Blon_0993 | hypothetical protein |
| Blon_0994 | transcriptional regulator, Fis family |
| Blon_1037 | conserved hypothetical protein |
| Blon_1494 | hypothetical protein |
| Blon_1495 | conserved hypothetical protein |
| Blon_1541 | hypothetical protein |
| Blon_1664 | GCN5-related N-acetyltransferase |
| Blon_1687 | TfoX, C-terminal domain protein |
| Blon_1688 | transcription activator, effector binding |
| Blon_1693 | two component transcriptional regulator, LuxR family |
| Blon_1698 | protein of unknown function UPF0102 |
| Blon_1950 | hypothetical protein |
| Blon_1951 | UMUC domain protein DNA-repair protein |
| Blon_2061 | extracellular solute-binding protein, family 1 |
| Blon_2064 | transcriptional regulator, DeoR family |
| Blon_2082 | lipopolysaccharide biosynthesis |
| Blon_2191 | ribose 5-phosphate isomerase |
| Blon_2370 | glycerophosphoryl diester phosphodiesterase |
| Blon_2371 | Glutamate--tRNA ligase |
| Blon_2372 | ATPase AAA-2 domain protein |
| dnaK | chaperone protein DnaK |
| groEL | chaperonin GroEL |
| recA | recA protein |
